# Nanoparticle-facilitated functional and molecular imaging for the early detection of cancer

**DOI:** 10.3389/fmolb.2014.00015

**Published:** 2014-10-17

**Authors:** Maharajan Sivasubramanian, Yu Hsia, Leu-Wei Lo

**Affiliations:** ^1^Institute of Biomedical Engineering and Nanomedicine, National Health Research InstitutesZhunan, Taiwan; ^2^Institute of Biotechnology, National Tsing Hua UniversityHsinchu, Taiwan

**Keywords:** nanoparticle, molecular imaging, cancer early diagnostics, iron oxide nanoparticles, gadolinium, quantum dots, polymeric nanoparticles, gold nanoparticles

## Abstract

Cancer detection in its early stages is imperative for effective cancer treatment and patient survival. In recent years, biomedical imaging techniques, such as magnetic resonance imaging, computed tomography and ultrasound have been greatly developed and have served pivotal roles in clinical cancer management. Molecular imaging (MI) is a non-invasive imaging technique that monitors biological processes at the cellular and sub-cellular levels. To achieve these goals, MI uses targeted imaging agents that can bind targets of interest with high specificity and report on associated abnormalities, a task that cannot be performed by conventional imaging techniques. In this respect, MI holds great promise as a potential therapeutic tool for the early diagnosis of cancer. Nevertheless, the clinical applications of targeted imaging agents are limited due to their inability to overcome biological barriers inside the body. The use of nanoparticles has made it possible to overcome these limitations. Hence, nanoparticles have been the subject of a great deal of recent studies. Therefore, developing nanoparticle-based imaging agents that can target tumors via active or passive targeting mechanisms is desirable. This review focuses on the applications of various functionalized nanoparticle-based imaging agents used in MI for the early detection of cancer.

## Introduction

Cancer is one of the world's most debilitating diseases, and its mortality rates continue to increase. More than 16 million new cancer cases are registered every year, and the medical expenses for overall clinical cancer treatment are practically unaffordable for most patients (Jemal et al., 2011; Morgan et al., 2011; Siegel et al., 2014). These increases in cancer mortality rates are due to a lack of early detection and subsequent treatment. Biomedical imaging techniques, such as magnetic resonance imaging (MRI), optical imaging (OI), X-ray computed tomography (CT) and ultrasound play crucial roles in clinical oncology (Weissleder, 2002; Juweid and Cheson, 2006; Fass, 2008) (Table 1). These imaging methods can provide essential information regarding tumor location, size, and distribution at high spatial resolution. The information obtained from these imaging modalities greatly assists clinicians in treatment strategy selection, which is beneficial for patients. Unfortunately, conventional biomedical imaging techniques detect cancerous tumors when they are more than a centimeter in diameter; at this stage, the lesion already consists of more than 10^9^ cells. Here, molecular imaging (MI) is likely to serve a pivotal role because it enables visualization of important biological processes that are associated with the early stages of carcinogenesis in a detailed and specific manner using targeted imaging agents. MI is more sophisticated than conventional bioimaging because the contrast agents consist of tumor-specific ligands, such as antibodies or peptides. These agents may also be small molecules or signaling moieties, such as fluorophores or radionuclides, or paramagnetic metal chelates. A typical contrast agent is expected to accumulate selectively at a desired site and interact physically or chemically with a target, thereby altering the imaging contrast. Hence, MI serves an integral role in the early detection of cancer and in drug discovery and development (Rudin and Weissleder, 2003; Jaffer and Weissleder, 2005; Weissleder, 2006; Willmann et al., 2008; Pysz et al., 2010; Hussain and Nguyen, 2014).

**Table 1:** **Advantages and disadvantages of imaging modalities used with targeted or non-targeted imaging agents**.

**Modality**	**Advantages**	**Disadvantages**	**Contrast agents**	**Radiation**
CT	Unlimited tissue depth penetration	Radiation exposure	Krypton	X-rays
	High spatial resolution	Poor soft tissue contrast	Xenon	
	Can be used for whole body imaging		Iodine	
	Low acquisition time		Barium	
	Anatomical imaging			
Positron emission tomography (PET)	Unlimited tissue depth penetration	Radiation exposure	^11^C	High energy γ-rays
	Can be used for whole body imaging	Low spatial resolution and long acquisition time	^18^F	
	Detailed anatomical information can be obtained when combined with CT or MRI	Expensive	^64^Cu	
MRI	Unlimited tissue depth penetration	Expensive	Gd ^3+^	Radio waves
	Non-ionizing radiation	Long acquisition time and limited sensitivity	SPIO, USPIO	
	High spatial resolution and excellent soft tissue contrast			
	Whole body imaging possible			
Ultrasound	High spatial resolution	Cannot be used for whole body imaging	Microbubbles	High frequency sound
	Real time imaging with low acquisition time			
	Highly sensitive and inexpensive			
OI	High spatial resolution	Cannot be used for whole body imaging limited tissue depth penetration	Fluorescent dyes and molecules Light sensitive NPs	Visible or near-infrared light
	Real time imaging with low acquisition time			
	Highly sensitive and inexpensive			

In general, targeted organic fluorophores and radioisotopes are commonly used as contrast agents in MI (Ballou et al., 1995; Becker et al., 2001; Massoud and Gambhir, 2003; Pham et al., 2004; Schnell et al., 2009). Contrast agents must possess long circulation times and the ability to reach the desired target site at an adequate concentration, which is highly essential for their successful detection *in vivo*. Unfortunately, clinical applications of contrast agents are severely limited due to their short half-lives, their ability to elicit an immune response and the difficulty that they experience with crossing biological membranes. As a result, only a few imaging agents (For example, Feridex®, Magnevist®, Gadavist®, AK Fluor®, Fluorescite®, IC-Green®, etc.) approved by Food and Drug Administration (FDA, USA) are available for clinical use.

**Figure 1:**
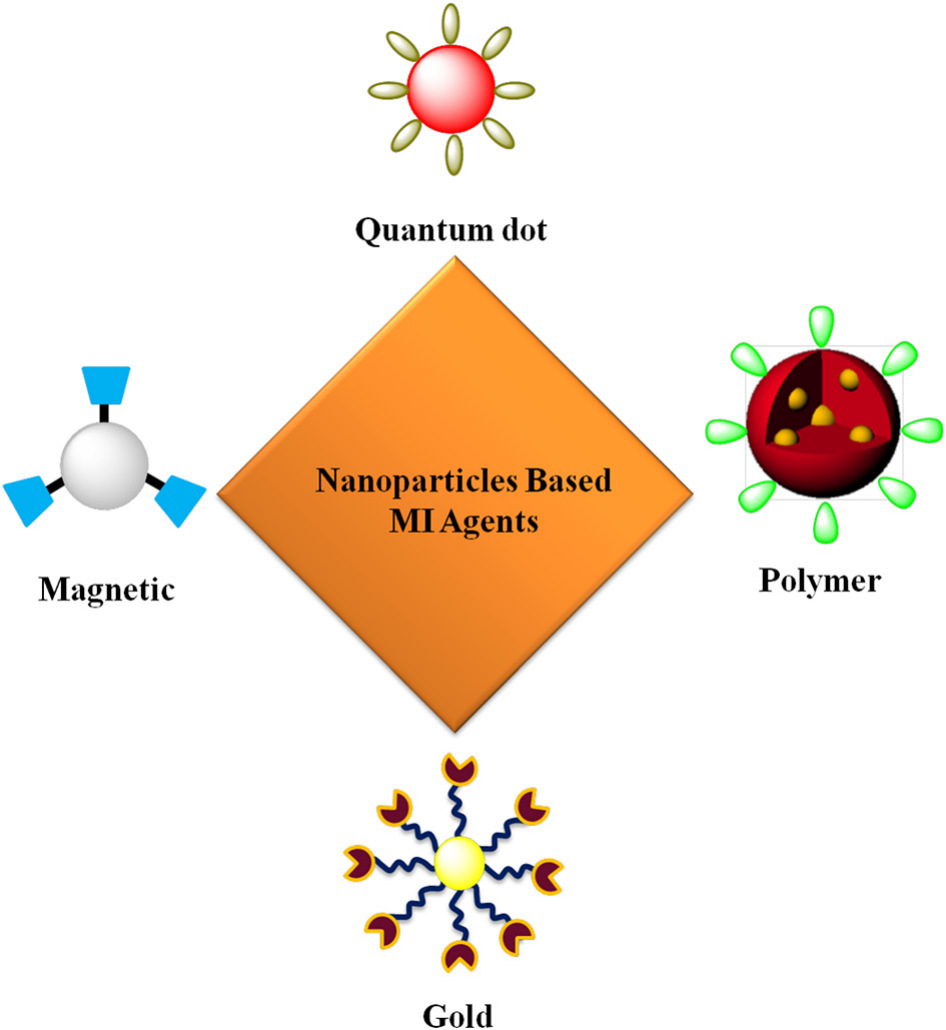
**Targeted nanoparticles for MI**.

**Figure 2:**
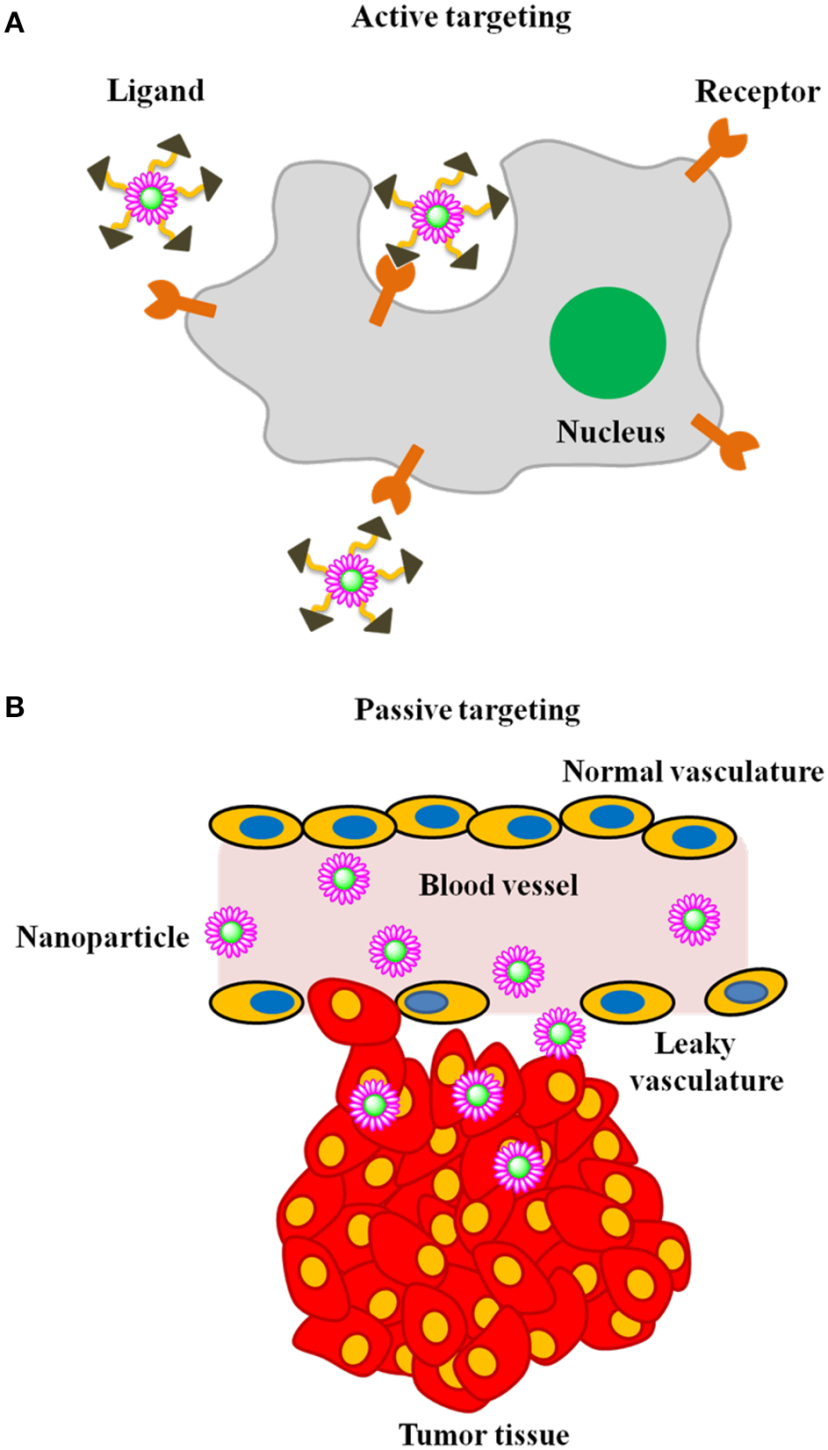
**Illustration of mechanism of tumor targeting (A) active targeting and (B) passive targeting**.

In recent years, nanoparticles have gained significant attention as contrast agents for the early detection of cancer (Figure 1). Using nanoparticles, it is possible to (1) achieve high specificity toward a target, thereby eliminating the danger of side effects; (2) deliver a large payload amount in a single dose and (3) simultaneously deliver both imaging agents and therapeutics. Carefully designed nanoparticle-based contrast agents (NCAs) could overcome biological barriers and reach tumors through either active or passive targeting mechanisms (LaVan et al., 2003; Ferrari, 2005; Couvreur and Vauthier, 2006; Peer et al., 2007) (Figure 2). In passive targeting, a distinguishing feature of a tumor enables an NCA to accumulate and be retained in the tumor via the enhanced permeation and retention (EPR) effect. In contrast, active targeting involves the conjugation of targeting ligands to the surface of the NCA, enabling the NCA to enter the cancer cell via receptor-mediated endocytosis (Saravanakumar et al., 2009; Choi et al., 2010; McCarthy et al., 2010). The aim of this review is to highlight various NCAs used for early cancer detection with MI.

## MRI

MRI uses the nuclear magnetic resonance signals of hydrogen atoms in water to generate images. After a brief radiofrequency pulse, the rate at which protons relax back to their equilibrium state, dependent on their physiochemical environment, gives rise to an MRI signal. MRI holds great potential as a clinical imaging and diagnostic tool due to its excellent soft tissue contrast, high spatial resolution, and ability to deeply penetrate tissues (Stark and Bradley, 1999; Jacobs and Cherry, 2001; Sosnovik and Weissleder, 2007). The most commonly used contrast agents (T_1_ and T_2_) for clinical cancer diagnosis are gadolinium chelates and superparamagnetic iron oxide nanoparticles (SPIOs) (McCarthy and Weissleder, 2008; Islam and Josephson, 2009; Lin et al., 2009; Cheng et al., 2013; Zhou and Lu, 2013). Bare SPIOs are used in the clinic to image liver tumors and metastases with sharp contrast, providing clear distinction between normal and cancerous tissues. This diagnostic ability arises from the fact that SPIOs are readily taken up by reticuloendothelial cells (Kupffer cells), which are absent from or present in lower concentrations in tumors (Saini et al., 1987; Lin et al., 2009). SPIOs with diameters of 30 nm are known to accumulate in lymph nodes by interstitial–lymphatic fluid transport and can be consequently used to detect lymph node metastases (Anzai et al., 2003). In addition, ultrasmall SPIOs (USPIOs) have been investigated as contrast agents for improving the delineation of brain tumors on MR images (Enochs et al., 1999; Neuwelt et al., 2004).

### Iron oxide nanoparticles

To improve tumor detection and localization, SPIOs are chemically modified with ligand molecules that target specific upregulated receptors or proteins and serve as MI probes. Yang et al. developed functionalized iron oxide nanoparticles (IOs) to target urokinase-type plasminogen activator receptor (uPAR). uPAR is well known to be upregulated in a variety of tumor cells and tumor-associated endothelial cells, such as fibroblasts and macrophages (Han et al., 2005). High uPAR expression is closely associated with metastasis and tumor invasiveness; hence, uPAR is an excellent target for imaging breast cancer cells. In this study by Yang et al., IOs were coated with a carboxylic acid-bearing amphiphilic polymer. The large number of carboxylic groups on the surface allowed covalent modification with poly(ethylene glycol) (PEG) and uPA bearing Cy5.5. Confocal microscopy experiments demonstrated effective uptake of the targeted nanoparticles by uPA receptors, which were overexpressed on mouse mammary tumor 4T1 cells, whereas human breast cancer T47D cells that lacked uPA receptors did not uptake the nanoparticles. Biodistribution studies in 4T1 tumor-bearing mice demonstrated significant accumulation of the nanoagent in the tumors and showed increased fluorescence signal intensity for up to 48 h. Similar tumor-targeting properties of these nanoagents were also observed using MR imaging in a mouse model (Yang et al., 2008). In another study, Yang et al. used the amino-terminal fragment (ATF) of the high affinity receptor binding domain of uPA-conjugated IOs to target uPAR. The results of this study showed that ATF-conjugated IO specifically bound to uPAR and was taken up by the cells. *In vivo* systemic delivery of ATF-IO nanoparticles showed selective accumulation in orthotopic pancreatic tumors, as demonstrated by both OI/MR imaging. *In vivo* OI clearly showed strong near infrared signals in the pancreatic tumors; however, no signal was detected in the liver or spleen of the tumor-bearing mice that received Cy5.5-ATF-IO nanoparticles. Additionally, the targeted nanoparticles exhibited high sensitivity and were able to detect tumors as small as 0.5–1 mm^3^ in animal tumor models using MRI. The authors suggested that the targeted nanoparticles used in this study have the potential for early detection of pancreatic cancer lesions by MRI and for intraoperative delineation of tumor margins and peritoneal metastases by OI (Yang et al., 2009a).

Chen et al. developed herceptin (HP)-conjugated cross-linked iron oxide nanoparticles (CLIOs) for breast cancer imaging. HP is a monoclonal antibody that has the ability to bind HER-2/neu receptors, which are overexpressed in breast cancer cells (Tran et al., 2007). Primary amine-functionalized CLIOs were prepared by cross-linking dextran-coated SPIOs with 2,2′-(ethylenedioxy)bisethylamine, followed by chemical conjugation with HP. *In vitro* studies demonstrated efficient uptake of targeted CLIOs in HER-2/neu receptor-overexpressing cancer cells (SKBr3, MDA-MB-231, and MCF-7); uptake was not observed in KB cells that lacked HER-2/neu receptors. From *in vivo* MRI studies, the results showed that when administered intravenously in mice bearing SKBr3 and KB tumors, targeted CLIOs selectively accumulated in the SKBr3 tumors but not in the KB tumors, as confirmed by the darkening of the tumors in T_2_-weighted images (Chen et al., 2009). Li et al. developed octreotide peptide (OP)-modified USPIOs to target somatostatin receptors (SSR) in breast cancer cells. Somatostatin (ST), a polypeptide known as somatotropin release inhibiting factor (SRIF), exhibits an affinity for the receptors overexpressed in neuroendocrine tumors and on cells of the immune system. OP is an analog of ST that has the ability to bind several SSR subtypes. To prepare SSR-targeted contrast agents, one end of dicarboxylated PEG was attached to USPIOs, and the other end was conjugated to OPs. *In vitro* MR imaging results revealed that the targeted nanoparticles displayed significantly lower T_2_ signal values, suggesting efficient uptake by breast carcinoma MCF-7 cells compared to non-targeted nanoparticles. For *in vivo* MR imaging studies, both targeted and non-targeted nanoparticles were administered to MCF-7 tumor-bearing mice. The results showed that the targeted nanoparticles accumulated in the tumor, which was confirmed by the observed signal decrease in T_2_-weighted images. In addition, non-targeted nanoparticles also exhibited a small decrease in signal; this might be due to random accumulation in the tumor by the EPR effect (Li et al., 2009). Luteinizing hormone-releasing hormone (LHRH) is known to bind LHRH receptors that are overexpressed on breast cancer cell membranes (Leuschner et al., 2005). Hence, LHRH is a suitable breast tumor-targeting molecule. Meng et al. developed LHRH receptor-targeted SPIOs to image breast tumors. *In vitro* and *in vivo* studies in breast cancer cells demonstrated the use of targeted nanoparticles as tumor-specific T_2_ contrast agents in relevant models. The cell uptake behavior of targeted SPIOs was much greater than that of bare SPIOs. The increased uptake and intracellular accumulation of targeted SPIOs provided enhanced T_2_ contrast with improved spatial resolution. The improved targeting by this nanoagent may also be used for cancer therapy via loading the nanoparticles with therapeutic drugs (Meng et al., 2009).

Kelly et al. developed a new peptide (IPL) to image prostate cancer. IPL has the ability to bind a type II transmembrane serine protease called hepsin (HPN), which is uniquely expressed in prostate cancer cells. In this study, IPL was conjugated to a bimodal nanoparticle that bore an optically detectable fluorophore attached to IOs. In a mouse xenograft model, HPN-targeted nanoparticles exhibited high specificity for HPN-overexpressing LNCaP xenografts compared to non-HPN-expressing xenografts. The authors proposed that HPN-targeted nanoparticles could be used for the early detection of prostate cancer (Kelly et al., 2008). Kresse et al. developed transferrin (Tf)-conjugated USPIOs for targeting malignant tumors. Tf is an iron-transporting serum glycoprotein that has the ability to bind specific receptors that are overexpressed in various cancer cells (Hopkins and Trowbridge, 1983). *In vivo* MR imaging studies in SMT/2A tumor-bearing rats showed that targeted USPIOs exhibited substantial signal reduction (40%) in T_2_-weighted MR images. In addition, control experiments using bare USPIOs or non-specific human serum albumin-conjugated USPIOs also accumulated in the tumors with a 10% change in the MR signal intensity, most likely due to non-specific accumulation in the tumors (Kresse et al., 1998). Choi et al. used folic acid (FA)-conjugated magneto-fluorescent nanoparticles as a targeted imaging agent for cancer detection. FA is essential for cell proliferation and for the maintenance of new cells during rapid cell division. Folate receptors are frequently upregulated in various cancer cells, enabling cancer cells to access vitamins necessary for their survival (Antony, 1996; Lu and Low, 2002). Hence, folate-targeted imaging probes are useful for the detection of cancer cells that overexpress folate receptors. *In vitro* confocal observation showed a strong fluorescence signal in folate receptor-overexpressing KB cells incubated with the targeted nanoagent, whereas no fluorescence signals were detected in cells (human lung carcinoma) lacking folate receptors. An *in vivo* MR imaging study in a mouse KB tumor xenograft model showed that the targeted nanoagent exhibited an average intensity decrease of 38% from pre- to post-contrast images of the tumor, which was approximately three times the intensity decrease observed in a non-tumor-bearingmouse. These targeted nanoparticles are highly specific and can be used as targeted delivery systems for tumor diagnosis and therapy (Choi et al., 2004).

For targeting brain tumors, Meng et al. developed SPIOFCs, a dual imaging nanoparticle consisted of chlorotoxin (CTX) and fluorescein isothiocyanate (FITC) conjugated to SPIOs. CTX, a small peptide found in the deathstalker scorpion (Leiurus quinquestriatus), is a highly specific marker for glioma cells and has been used for brain tumor targeting in animal models (Soroceanu et al., 1998). *In vitro* studies demonstrated that prepared SPIOFCs did not induce any toxicity and showed increased cellular uptake in human U251-MG and rat C6 glioma cells when evaluated by MR imaging and inductively coupled plasma emission spectroscopy (ICPMS). Glioma cells were found to be insensitive to bare nanoparticles without CTX and showed no significant uptake; these findings suggested that the presence of CTX was essential for specific and efficient targeting of glioma cells (Meng et al., 2007). Another study by Sun et al. used CTX-conjugated PEGylated iron oxide nanoparticles (CPIs) as an MI probe to detect gliomas. In general, the presence of PEG in nanoparticles offers stealth properties and allows the conjugation of various molecules, such as diagnostic, targeting, and therapeutic agents. *In vitro* MR imaging showed increased cellular uptake of CPI nanoparticles in 9L gliosarcoma cells compared to non-targeted nanoparticles. In athymic (nu/nu) mice bearing 9L gliosarcoma xenograft tumors, CPIs gradually accumulated in the tumors over time as expected, and small amounts of non-targeted nanoparticles also reached the tumors through non-specific mechanisms. The contrast enhancement degree was quantified by measuring the R_2_ relaxation rates (Sun et al., 2008). For the specific identification of apoptotic cells, Schellenberger et al. chemically conjugated CLIOs to annexin-V, a protein that binds to extracellular phosphatidylserine, an early marker of apoptosis (Martin et al., 1995). In this study, Jurkat T lymphoma cancer cells were used, it is well known that insufficient apoptosis is one of the characteristic features of cancer cells. For specific identification, apoptosis was induced in Jukart cells using camptothecin, an anticancer drug. From *in vitro* MRI studies, it was found that targeted CLIOs identified camptothecin-induced apoptosis of Jurkat T cells, which was confirmed by the significant signal decrease relative to untreated cells; in contrast, bare CLIOs failed to induce significant changes in apoptotic cells. These results suggest that the targeted CLIOs used in this study can serve as an MRI probe for imaging apoptosis *in vivo* (Schellenberger et al., 2002).

#### Clearance of IOs

In general, intravenously administered IOs are cleared from blood circulation by reticulo endothelial system (Corot et al., 2006). However, the various properties such as shape, size, and surface characteristics largely determine the pharmacokinetics and clearance. For instance, IOs with larger size are eliminated faster than smaller-sized NPs. Jain et al. investigated the biodistribution, clearance, and biocompatibility of IOs sequentially coated with oleic acid and pluronic (Pluronic-OA-IO). Usually, IO formulations use dextran or starch coating as a stabilizing agent to form a water-dispersible system. In this study, pluronic or PEG were used as coating materials, which are known to prevent opsonization. *In vivo* biodistribution studies of Pluronic-OA-IO (193 nm) showed about 55% of the injected iron accumulated in the liver. A gradual increase in the serum iron level was observed for 1 week, which indicated that clearance of nanoparticles require more than 3 weeks (Jain et al., 2008).

Lewis et al. studied the biodistribution of Ferumoxtron-10, a USPIO (30 nm in diameter) coated with low molecular weight dextran. Ferumoxtran-10 demonstrated slow clearance of iron and was eliminated (89%) in the urine in 7 weeks. Iron contained in Ferumoxtron-10 was absorbed into the body's iron store and gradually into hemoglobin. Like endogeneous iron, it was eliminated slowly (16–21%) after 84 days through hepatobiliary mechanism (Bourrinet et al., 2006).

### Gadolinium-based nanoparticles

Paramagnetic gadolinium (Gd) complexes are the most widely used T_1_ contrast agents for clinical applications of MRI (Caravan et al., 1999; Lee et al., 2014). In recent years, many researchers have investigated the use of several targeted Gd complexes for the early detection of cancer (Damadian, 1971; Zhou and Lu, 2013; Hussain and Nguyen, 2014). The use of Gd complexes as positive contrast agents in T_1_-weighed MRI sequences is limited by their sensitivity. Hence, Gd complexes are usually encapsulated in nanocarriers or are chemically conjugated to synthetic polymers, such as dendrimers, to amplify their sensitivity (Kobayashi and Brechbiel, 2003, 2005; Luo et al., 2011). For targeting purposes, Gd complexes are covalently conjugated to targeted ligands, which dock and respond to specific receptors that are overexpressed in cancer cells.

**Figure 3:**
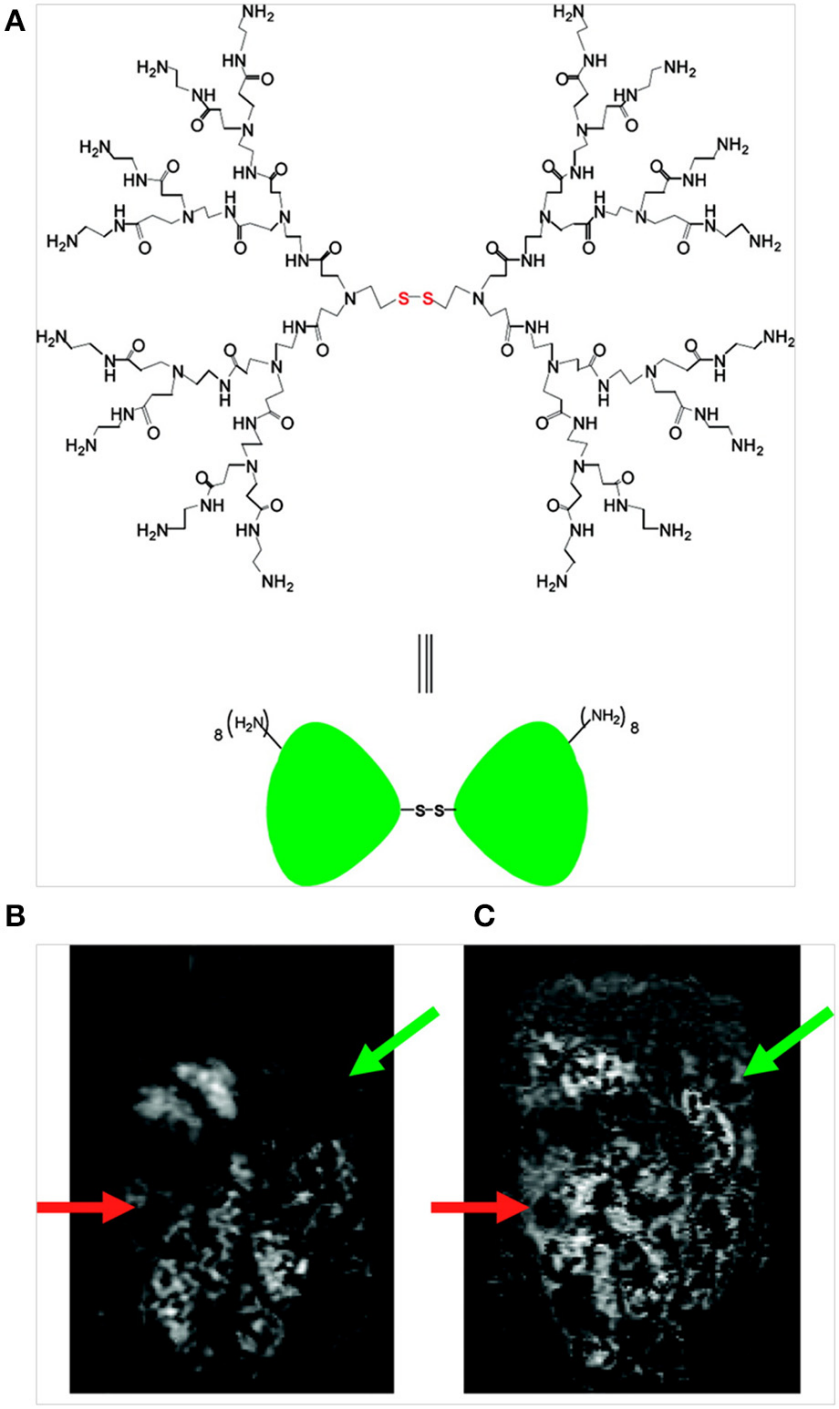
**(A)** Structure of a PAMAM dendrimer possessing a disulfide cystamine core; **(B)** T_1_-weighed MR image obtained prior to contrast agent injection (*t* = 0) reveals an area of tumor growth below the liver, stomach and spleen in the left abdomen (green arrow) and adjacent to the gastrointestinal tract in the lower right abdomen (orange arrow) and **(C)** T_1_-weighted MR image obtained 6.5 h after intra-peritoneal injection of the dual contrast agent (green and orange arrows indicate signal enhancement on the tumor surface) (Xu et al., 2007), © 2007, American Chemical Society.

Dendrimers are self-assembled polymer constructs with tunable surface and are widely used as carriers for Gd-based contrast agents. Xu et al. developed a bimodal second-generation (G2) dendrimer nanoparticle that consisted of covalently conjugated Gd-1B4M- diethylenetriaminepentaacetate (DTPA) and optically detectable rhodamine green for targeting ovarian cancer (Figure 3). The nanoparticles achieved success in reaching tumor tissues and delivered significant amounts of imaging payloads in an ovarian tumor-bearing mouse xenograft model to produce detectable changes in the tumors by optical and MRI (Xu et al., 2007). Regino et al. evaluated the use of the dendrimer-based contrast agent Gd-G8 as a dual MRI and CT contrast agent for precisely monitoring convection-enhanced delivery to the brain. From *in vitro* studies, CT-based attenuation values of the Gd-based agent were ~1.6 times greater than those of iopamidol (an iodine-based agent); moreover, the attenuation of the Gd-G8 dendrimer was similar to that of Gd-DTPA. *In vivo* studies demonstrated that detectable signal enhancement could be observed by CT and MRI with an optimal dose of Gd-G8 over a range of 23–78 mM; however, for the adequate delineation of the injection site on MRI and CT, a concentration of at least 47 mM was required, and the distribution volume (Vd) was estimated with greater sensitivity by MRI than by CT (Regino et al., 2008).

Swanson et al. prepared a target-specific multifunctional nanoparticle, Gd(III)-DOTA-G5-FA that consisted of Gd-complexed, 1,4,7,10-tetraazacyclododecane-1,4,7,10-tetraacetic acid (DOTA) and FA conjugated to a G5 poly (amidoamine) (PAMAM) dendrimer. *In vivo* studies were performed in a NODCB-17 SCID mouse xenograft model with KB tumors. When administered subcutaneously, the Gd(III)-DOTA-G5-FA dendrimer nanoparticles specifically reached the tumors and generated statistically significant MRI signal enhancement compared to non-targeted counterparts (Gd(III)-DOTA-G5). Sustained signal enhancements in the tumors were observed even at 48 h post-injection, suggesting the effective tumor-targeting ability of Gd(III)-DOTA-G5-FA (Swanson et al., 2008). Huang et al. prepared multifunctional dendrigraft poly-L-lysines (D2 and D3) composed of covalently attached CTX and Gd-DTPA. The OI results showed that CTX dendrimer conjugates were readily taken up by C6 glioma and liver tumor cell lines but were not taken up by normal cell lines. Intracranially administered CTX dendrimer conjugates in C6 glioma-bearing mice preferentially accumulated in the brain tumors, suggesting their tumor-targeting ability. In addition, the MRI results demonstrated substantial signal enhancement in mice treated with CTX-modified contrast agent, which reached a maximum level at 5 min for both gliomas and liver tumors (Huang et al., 2011).

Olson et al. developed a G5 PAMAM dendrimer coated with activatable cell-penetrating peptides (ACPPs) bearing Cy5 or Gd or both for the dual monitoring of matrix metalloproteinase (MMP) activity. The uptake of ACPP-conjugated nanoparticles in a human fibrosarcoma cell line was 4- to fifteen-fold higher than that of bare nanoparticles. Residual tumors and metastases as small as 200 μm were successfully detected using OI. In the presence of protease, Gd-labeled nanoparticles released high levels of Gd (30–50 μm) to the tumor parenchyma, which resulted in enhanced monitoring using T_1_-weighted contrast for a longer period after injection (Olson et al., 2010).

Inadequacies in the ability to image and treat malignant gliomas are mainly due to the blood-brain barrier. Sarin et al. developed several Gd-DTPA-functionalized PAMAM dendrimers of various sizes for transvascular delivery across the blood-brain barrier. When administered intravenously in RG-2 glioma-bearing mice, Gd- and fluorescent dye-functionalized dendrimers (11.7–11.9 nm) effectively crossed the blood-brain barrier and accumulated in the tumors. In this study, the authors found that the physiological upper limit of nanoparticle size for traversing the blood-brain barrier was less than 11.7–11.9 nm (Sarin et al., 2008). This investigation also showed that in the physiological state, a fibrous glycocalyx covers the luminal surface of the blood-tumor barrier (BTB) of both the brain tumor and the peripheral tumor microvasculature, which is the major impediment for the transport of particles. Hence, the authors indicated that the peripheral tumor vasculature is highly permeable to macromolecules smaller than approximately 12 nm due to the presence of a greater number of pores underlying the glycocalyx of the BTB (Huang et al., 2011). Nanoglobular Gd-DOTA conjugates [lysine dendrimers (G2 and G3) with cubic silsesquioxane cores] were attached to the peptide CLTI, which specifically binds to fibrin-fibronectin complexes that are present in the tumor extracellular matrix (Pilch et al., 2006), and were subsequently used for MRI identification of tumor tissues in female nude mice bearing MDA-MB-231 human breast carcinoma xenografts. The T_1_ relaxivities of the targeted G2 and G3 nanoglobules were 7.92 and 8.20 mM^−1^s^−1^, respectively, and the nanoglobules exhibited greater contrast enhancement in tumor models than corresponding non-targeted contrast agents (Tan et al., 2014). In another study, a Gd-DTPA-PAMAM-PEG dendrimer was conjugated to T7, a peptide that has an affinity for Tf receptors expressed on gliomas and liver cancer cells (Han et al., 2010, 2011a). T7-conjugated Gd-DTPA-PAMAM-PEG dendrimers (T7- GdDTPA-PAMAM-PEG) were then used for MRI identification of tumors in human hepatocarcinoma Bel-7402- and glioma C6-bearing mice. T7-GdDTPA-PAMAM-PEG exhibited an excellent ability to target tumors in hepatocarcinoma-bearing mice; moreover, the accumulation of T7-GdDTPA-PAMAM-PEG was 162.5% times that of PAMAM-PEG. However, the peptide conjugate was insensitive to gliomas (Han et al., 2011b). Multifunctional nanoparticles carrying Gd-DTA and FA conjugated to cross-linked G5 PAMAM dendrimers (dendrimer nanoclusters) were developed for tumor-targeted MRI. FITC-labeled dendrimer nanoclusters were readily taken up by folate receptor-expressing KB cancer cells, and in the presence of free FA, cell uptake was significantly reduced, suggesting receptor-mediated uptake. In mice bearing KB xenograft tumors, subcutaneously administered dendrimer nanoclusters gradually accumulated in the tumors; this accumulation was detected with a strong MRI signal. The incorporation of FA in the dendrimer nanoclusters established tumor specificity, which resulted in enhanced images (Cheng et al., 2010).

## OI

OI is a non-invasive technique that can be broadly divided into fluorescence and bioluminescence imaging. Fluorescence imaging is based in principle on using external light energy of an appropriate wavelength to excite a fluorophore; the excited fluorophore then emits a light of longer wavelength with lower energy. In contrast, bioluminescence imaging involves a chemical reaction between target and substrate molecules to generate luminescence, which is detected externally as evidence of a molecular process (Luker and Luker, 2008; Weissleder and Pittet, 2008; Willmann et al., 2008). The main challenges to OI are limited tissue penetration and tissue autofluorescence. Light in the visible spectrum is strongly absorbed by hemoglobin and other biomolecules, such as water and lipids, which significantly reduces the signal by ten-fold. As an alternative to light in the visible region of the spectrum, light in the near infrared (NIR) region (600–1000 nm) has been used to image fluorescence in deeper tissues because tissue autofluorescence is reduced significantly in the NIR region, and little absorption of light by the endogenous biomolecules occurs. Hence, tremendous effort has been invested in the development of NIR probes for OI.

### Quantum dots

Semiconducting fluorescent nanocrystals known as quantum dots (QDs) have been widely used as probes for biomedical imaging and labeling due to their unique optical properties. QDs exhibit broad absorption spectra, high quantum yields, low photobleaching, and resistance to chemical degradation compared to traditional fluorophores, such as organic dyes (Bruchez et al., 1998; Medintz et al., 2005; Michalet et al., 2005). For cancer-related molecular and cellular imaging applications, the surface of QDs is usually modified with biomolecules, such as antibodies, peptides or small molecules that have the ability to adhere and respond to receptors or antigens present on target cells (Zhang et al., 2008; Lee et al., 2010a; McCarthy et al., 2010; Cheng and Cheng, 2012; Pericleous et al., 2012). Substantial developments in nanotechnology and materials chemistry have encouraged scientists to engineer QDs to carry therapeutic agents for concurrent imaging and therapy.

**Figure 4:**
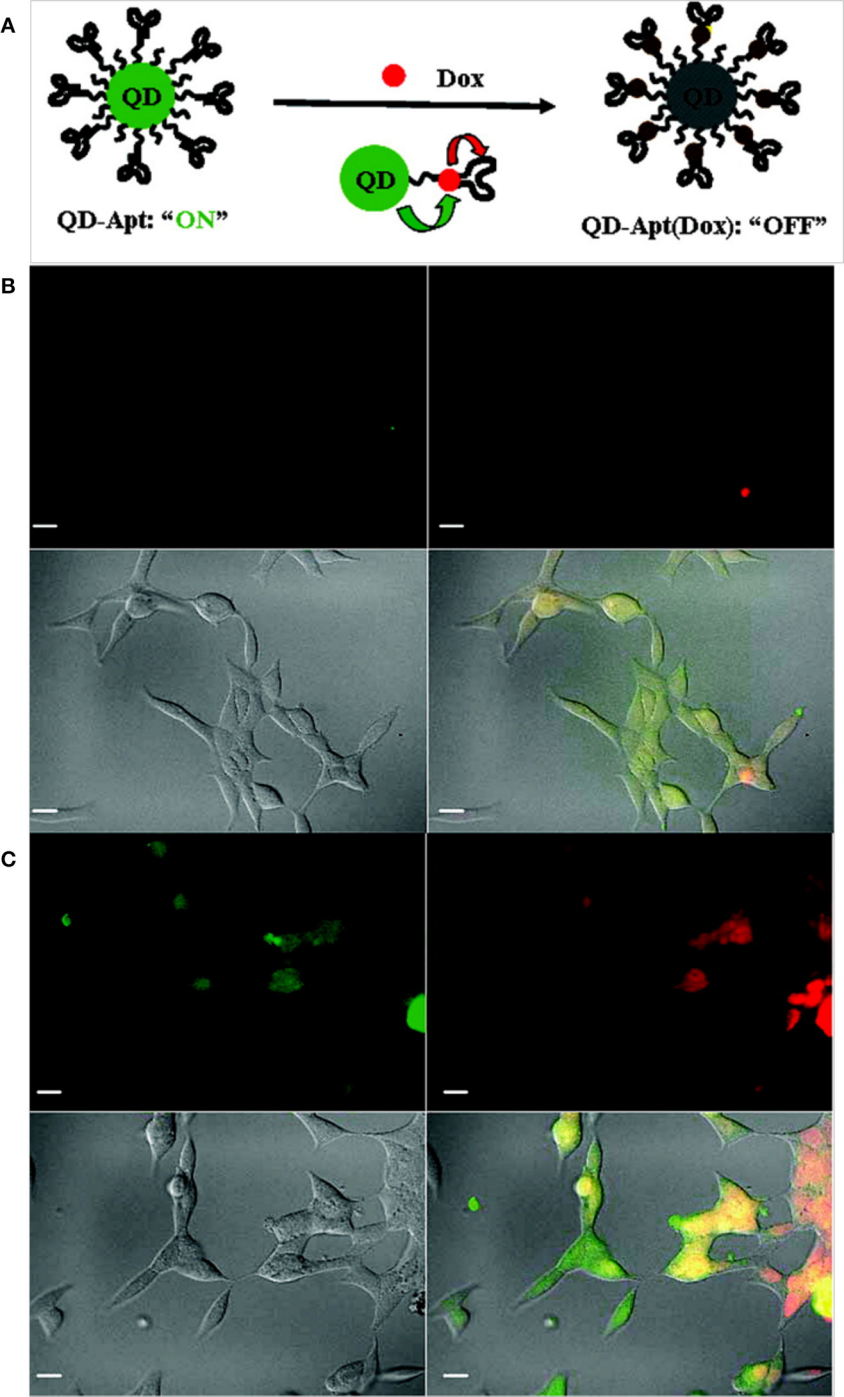
**(A)** A schematic illustration of QD-Apt(Dox) Bi-FRET. Confocal laser scanning microscopy images of QD-Apt DOX conjugates incubated in PSMA-expressing LNCaP cells at **(B)** 0 h and **(C)** 1.5 h. DOX and QDs are shown in red and green, respectively, and the lower right images in each panel represent an overlay of DOX and QD fluorescence (Bagalkot et al., 2007), © 2007, American Chemical Society.

Epidermal growth factor receptor (EGFR) is a transmembrane glycoprotein that is overexpressed in breast cancers and can be utilized as a biomarker for imaging (Herbst, 2004). Yang et al. developed anti-EGFR antibody-conjugated QDs for targeted breast cancer imaging. Initially, carboxylic acid-functionalized QDs were covalently linked to the primary amine of modified nickel (II) nitrilotriacetic acid (NNTA) via a stable amide bond. To target EGFR, a histidine-tagged anti-EGFR antibody (ScFv EGFR) was conjugated to the NNTA-QDs. *In vitro* studies clearly demonstrated that the targeted NNTA-QDs exhibited an affinity for EGFR-overexpressing breast cancer cells (4T1 and MDA-MB-231) and were readily internalized, whereas non-targeted NNTA-QDs showed no significant binding to breast cancer cells (Yang et al., 2009b). Gao et al. reported prostate-specific membrane antigen (PSMA)-bearing QD conjugates for targeted *in vivo* imaging of prostate cancer. Initially, QDs were sequentially coated with n-octylphosphine oxide and a carboxylic acid-bearing amphiphilic polymer. The polymer served to protect the QDs from enzymatic digestion and hydrolysis *in vivo*. Surface-coated QDs were then conjugated to PEG to prolong their circulation time *in vivo*. *In vitro* studies demonstrated that the targeted QDs were readily taken up by PSMA-overexpressing C4-2 human prostate cancer cells, while non-targeted PEGylated QDs exhibited minimal uptake by prostate cancer cells. When administered to prostate tumor-bearing nude mice, targeted QDs accumulated in the tumors with high specificity and exhibited a strong fluorescence signal, whereas no significant fluorescence signal was observed in mice injected with PEGylated QDs. Further studies, including biodistribution and histological analyses, clearly illustrated the prostate tumor-targeting potential of functionalized QDs (Gao et al., 2004). Bagalkot et al. developed QD-Apt, QDs functionalized with A10 RNA aptamer that recognizes the extracellular domain of PSMA for prostate cancer imaging, therapy and sensing. Doxorubicin (DOX) was able to bind to the double-stranded A10 RNA aptamer via non-covalent interactions, and the resulting conjugate (QD-Apt DOX) gained reversible self-quenching properties based on bi-fluorescence resonance energy transfer (Bi-FRET); in this process, QD-DOX and QD-Apt DOX acted as donor-acceptor pairs (Figure 4). Thus, the fluorescence of the QD-Apt DOX conjugate was quenched when intact, and the release of DOX from QD-Apt DOX inside of the cell allowed the nanoparticles to recover their fluorescence. When incubated with PSMA-overexpressing cancer cells (LNCaP), the nanoparticles were taken up and delivered DOX, which was confirmed by a strong fluorescence signal (Bagalkot et al., 2007).

**Figure 5:**
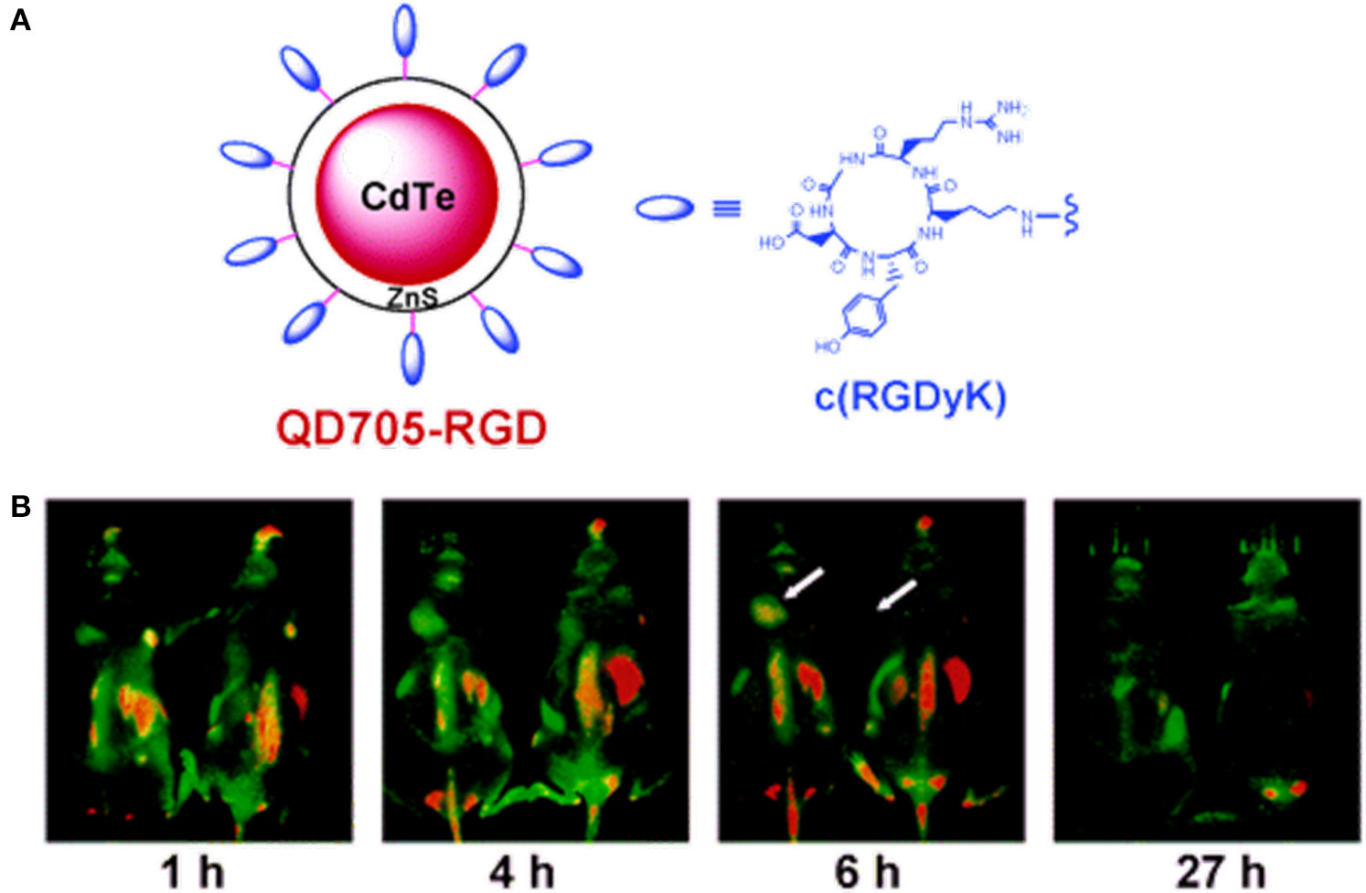
**(A)** RGD-bearing QD705 conjugates and **(B)** *in vivo* fluorescence imaging of tumor vasculature in a U87MG tumor-bearing mouse at various time intervals (Cai et al., 2006), © 2006, American Chemical Society.

Scroeder et al. developed lipodots, which consisted of QDs entrapped in a lipid shell and post-loaded with a folate-lipid conjugate for targeting tumors. *In vitro* confocal observations showed that the targeted lipodots were readily taken up by folate receptor-overexpressing cancer cells (J6456-FR and KB-FR), confirmed by a strong fluorescence signal, while non-targeted lipodots were found to be insensitive. *In vivo* studies with mice bearing J6456-FR tumors demonstrated the tumor-targeting ability of targeted lipodots following intra-peritoneal injection (Schroeder et al., 2007). Mulder et al. developed targeted bimodal nanoparticles (pQDs) that consisted of magnetically detectable PEGylated lipids that were covalently coupled to QDs bearing RGD (specific for α_*v*_β_3_ integrin interaction). When incubated with α_*v*_β_3_ integrin-overexpressing human umbilical vein endothelial cells (HUVECs), enhanced uptake of pQDs by HUVECs was observed compared to that of non-targeted pQDs. The optical properties of the pQDs were preserved, and they exhibited very high *r*1 relaxivity. The overall results suggested the ability of the nanoagent to target tumor vasculature (Mulder et al., 2005). In a similar study, Cai et al. evaluated the tumor vasculature-targeting ability of RGD-coupled QD 705 (QD705-RGD) (Figure 5). When administered intravenously in athymic nude mice bearing subcutaneous U87MG human glioblastoma tumors, QD705-RGD exhibited a significant ability to target tumor vasculature, and the tumor fluorescence intensity reached a maximum at 6 h post-injection. However, non-specific accumulation was also observed in the lymph nodes, liver, and bone marrow (Cai et al., 2006). Chang et al. prepared protease-activatable QDs that consisted of gold nanoparticles bound to QDs via a peptide sequence susceptible to degradation by enzyme proteases. When intact, the conjugate was optically quenched with a 71% reduction in luminescence; however, the conjugate became highly luminescent after the peptide linker was degraded by proteases. This type of probe could be useful for monitoring the biological activities of proteases (Chang et al., 2005). Smith et al. observed and recorded the binding of RGD-conjugated QD800 (QD with emission at approximately 800 nm) to tumor blood vessels expressing α_*v*_β_3_ in mice models. RGD-QD800 demonstrated significant uptake by tumors compared to control QDs (PEG-QD800 and RAD-QD800), suggesting the ability of RGD-QD800 to target tumors overexpressing α_*v*_β_3._ Intra-vitalmicroscopy observations in mice bearing an SKOV-3 ear tumor clearly showed that the targeted QDs did not target tumor cells but did target the tumor neovasculature. This observation was presumably due to their relatively large size (~20 nm in hydrodynamic diameter), which prevented the nanoparticles from extravasating into the tumor. The authors also found that RGD-QD800 adhered to their targets as aggregates rather than as individual nanoparticles (Smith et al., 2008). In an interesting work by Akerman et al., QDs were conjugated to three different peptides (GFE, F3, and Lyp-1) to demonstrate their specific targeting abilities. *In vivo* studies showed that QD-GFE (lung-targeting) conjugates accumulated in the lungs of mice following intravenous administration, whereas QD-F3 and QD-Lyp-1 conjugates specifically targeted blood vessels or lymphatic vessels in tumors. Unfortunately, the QDs were not sufficiently stable to remain luminescent in living cells and tissues. Hence, the QDs used in this investigation are not feasible for *in vivo* applications (Åkerman et al., 2002). In addition to RGD functionalization, QDs have been conjugated to several other peptides for tumor-specific targeting. Gao et al. designed and chemically synthesized anti-HER2 affibody molecules (Z HER2:342) for the specific targeting of HER2-expressing cells and tumors in imaging. Affibodies are small protein molecules that are engineered to specifically bind targets, such as other proteins or peptides. The synthesized Z HER2:342-QD conjugates exhibited tumor-targeting ability with superior imaging contrast in mice bearing HER2-overexpressing SKOV3 tumors compared to low-level HER2-expressing PC-3 mice. These results indicated that the Z HER2:342-QD conjugates were target-specific and had the potential to image HER2-overexpressing cells (Gao et al., 2011). Diagaradjane et al. prepared EGF-conjugated QDs that had the ability to distinguish EGF receptor-overexpressing tumors from nearby normal tissues. *In vivo* imaging exhibited three distinct phases for the EGF-QD nanoprobes: tumor influx (~3 min), clearance (~60 min) and accumulation (1–6 h). The prepared EGF-QDs exhibited optimal pharmacokinetics and superior docking ability to EGFR, which allowed quantifiable imaging of EGFR expression in human colorectal cancer xenografts in mice (Diagaradjane et al., 2008).

Qian et al. employed Tf and anti-claudin 4-conjugated CdSe/CdS/ZnS QDs for targeted imaging of pancreatic cancer. The targeted optical agent was stable and had superior photoluminescence efficiency, demonstrating pancreatic cell-specific uptake using a monoclonal antibody for anti-claudin 4. The targeting agent used in this study can be further tuned to image pancreatic cancer in its early stages (Qian et al., 2007). Yong et al. used anti-claudin 4- and anti-prostate stem cell antigen-bearing non-cadmium-based QDs (InP/ZnS QDs) as highly efficient, non-toxic targeted optical probes for imaging live pancreatic cancer cells. Antibodies, such as anti-claudin 4 and anti-prostate stem cell antigen, whose receptors are known to be upregulated in both primary and metastatic pancreatic cancers, were used as targeting agents. The targeted QDs demonstrated affinity and specific targeting for pancreatic cancer cell lines *in vitro*. In addition, the receptor-mediated delivery of targeted QDs was further confirmed by the observation of poor *in vitro* targeting in non-pancreatic cancer cell lines, which were negative for the claudin-4-receptor. These observations suggest that InP/ZnS QDs could be used as an efficient OI imaging nanoprobe in diagnostic imaging, particularly for early detection of pancreatic cancer (Yong et al., 2009).

#### Clearance of QDs

Choi et al. established the effect of hydrodynamic diameter (HD) and surface charge of QDs on their renal clearance. QDs with HD smaller than 5.5 nm demonstrated rapid and efficient urinary excretion. With the increase of HD from 4.36 to 8.65 nm, the serum half-life increased from 48 min to 20 h (25 fold increase), suggesting a positive correlation between serum half-life and HD. QDs with HD range of 4.36–5.52 was cleared form the body through renal mechanism, while QDs with large HD did not undergo renal clearance and was confined to the liver, lung and spleen. The anionic or cationic surface charge of QDs increased the HD by 15 nm due to the charge related adsorption by serum proteins, which decreased the renal clearance consequently. Whereas, zwitterionic coatings prevented the serum protein adsorption and demonstrated increased solubility and improved renal filtration (Choi et al., 2007).

Fischer et al. investigated the clearance of two different QD conjugates [mercaptoundecanoic acid (QD-LM) and bovine serum albumin (QD-BSA)] administered intravenously in rats. QD-BSA exhibited accelerated clearance (1.23 ± 0.22 mLmin^−1^kg^−1^) from the blood, which resulted in higher levels of liver uptake (99%) compared to QD-LM (40%). However, both conjugates were not detected in urine or feces during the entire time of experiment (10 days). This may be presumably due to large HD (25 nm for QD-LM and 80 nm for QD-BSA) (Fischer et al., 2006). Schipper et al. studied the clearance of two commercially available QDs with sizes 12 and 21 nm in diameter and effect of PEGylation on their clearance. The results showed that a 2000 mW PEG coating prolonged the blood half-life, but delayed accumulation in the liver. All particles were rapidly taken up and accumulated in the liver, whereas PEGylated particles accumulated in the bone, while unPEGylated particles did not (Schipper et al., 2007).

### Gold nanoparticles

The popularity of gold nanoparticles (AuNPs) in biomedical applications has increased significantly in recent years owing to their unique size-dependent properties. AuNPs are highly biocompatible, easy to prepare and generally regarded as non-toxic (Boisselier and Astruc, 2009). Their characteristic surface plasmon absorption property is responsible for their excellent optical properties, making them attractive candidates for labeling and imaging. Tailor-made AuNPs of different sizes, shapes and functionalized with various targeting molecules have been used for a number of applications in cancer therapy and imaging (Tong et al., 2009; Kumar et al., 2013). Due to their vast potential, several AuNP-based formulations are currently undergoing phase I and phase II clinical trials for cancer treatment (Thakor et al., 2011).

**Figure 6:**
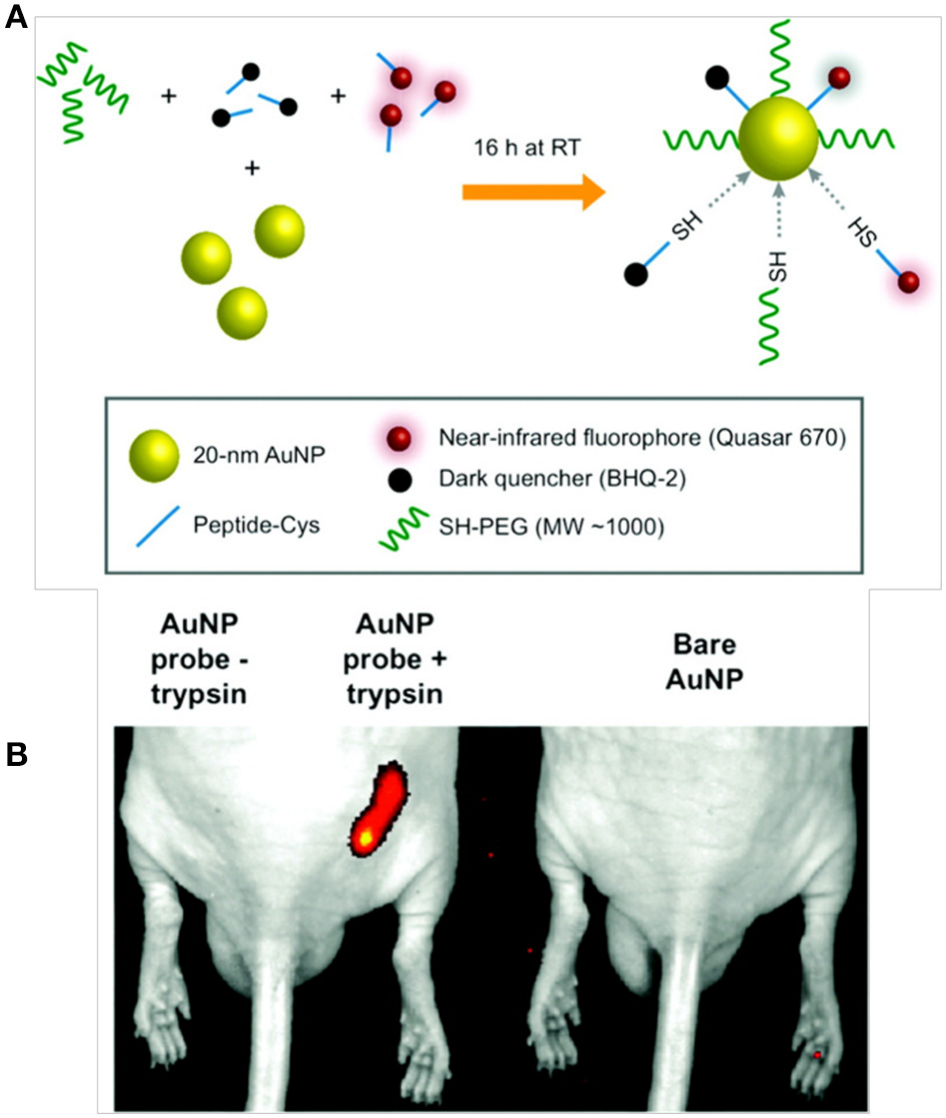
**Schematic diagrams of (A) AuNP probe synthesis and activation, and (B) *in vivo* near-infrared fluorescence imaging of subcutaneous tumor phantom models consisting of PuraMatrix peptide hydrogel mixed with AuNP probe in the presence or absence of trypsin as compared to unlabeled AuNPs (Mu et al., 2010), © 2010, American chemical society**.

The ability of AuNPs to quench fluorophores in close proximity encouraged Lee et al. to develop protease-sensitive NIRF probes for *in vivo* cancer imaging. In this study, AuNPs (20 nm) were stabilized by a Cy5.5-labeled MMP substrate, and the fluorescence of Cy5.5 was strongly quenched by the coordinated dye-dye and AuNP-surface quenching effects. When incubated with MMP *in vitro*, cleavage of the substrate allowed the probe to recover a strong fluorescence signal. In MMP-2-positive, SCC-7 tumor-bearing mice, the administered probes demonstrated preferential tumor accumulation with a strong fluorescence signal (Lee et al., 2008). Popovtzer et al. developed gold nanorods (AuNRs) conjugated to UMA9 antibodies for the targeted CT imaging of head and neck cancers. SCC represents more than 90% of all head and neck cancers, and UMA9 antibody can target SCC tumors *in vivo*. *In vitro* CT imaging revealed that targeted AuNRs were readily taken up by A9 antigen-overexpressing cancer cells (UM-SCC-1 and UM-SCC-5), which showed strongly selective X-ray attenuation that was distinct from that of normal cells (Popovtzer et al., 2008). Mu et al. developed AuNP-based activatable NIR probes functionalized with PEG and heterogeneous monolayers of dye-labeled peptides to monitor proteolytic activity *in vivo*. In this study, AuNPs (20 nm in diameter) were modified with a heterogeneous monolayer of fluorophore (Q670) and dark quencher (BHQ-2)-labeled peptide substrates that were responsive to proteases, such as trypsin and uPA (Figure 6). The resulting system demonstrated strong quenching properties due to the close proximity between Q670 and BHQ-2 to the AuNPs. In the presence of target enzymes *in vitro*, the nanoprobes recovered fluorescence with a five- to eight-fold increase in signal. In addition, AuNP-based probes exhibited prolonged circulation time *in vivo* with a t_1/2_ of more than 4 h and high image contrast in a mouse tumor model (Mu et al., 2010).

Chanda et al. developed bombesin (BBN) peptide-conjugated AuNPs to target gastrin-releasing peptide (GRP) receptors that are overexpressed on prostate cancer cells. BBN peptides exhibit a high affinity for GRP receptors that are overexpressed in prostate, breast and small-cell lung carcinomas *in vivo*. *In vitro* radioiodinated BBN displacement assays clearly demonstrated the GRP receptor specificity and binding affinity of the AuNP–BBN conjugates in PC-3 cells. *In vivo* CT imaging in prostate tumor-bearing, severe combined immune deficiency mice showed that intra-peritoneally administered AuNP–BBN conjugates preferentially accumulated in the tumors with little or no uptake in the liver relative to controls (Chanda et al., 2010). Kim et al. used multifunctional aptamer-AuNP conjugates for the combined targeted CT imaging of and therapy for prostate cancer. AuNPs were coated with thiol-functionalized capture oligonucleotides (ONT) that consisted of an A10 spacer and a (TCG)_7_ONT, which was hybridized with PSMA aptamer-(CGA)_7_ to create a double-stranded GC region on which several DOX molecules could be loaded. The targeted PSMA aptamer-AuNPs exhibited more than four-fold greater CT intensity in LNCaP cells that overexpressed PSMA antigen than in PSMA-negative PC3 cells. In addition, drug-loaded, aptamer-AuNPs demonstrated target-specific drug delivery to target cancer cells but not non-targeted cells (Kim et al., 2010). Chen et al. developed FA-conjugated bimodal G5 dendrimer nanoparticles carrying AuNPs loaded with Gd for targeted dual CT/MR tumor imaging. The multifunctional nanoprobes demonstrated the ability to image FA receptor overexpressing cancer models *in vitro* and *in vivo* (Chen et al., 2013).

Sun et al. reported tumor-targeting probes based on AuNPs for dual CT/OI of cancer. The targeted probe consisted of a Cy5.5-labeled, MMP-activatable peptide substrate that was chemically conjugated to glycol chitosan-coated AuNPs. The fluorescence of Cy5.5 was strongly quenched by BHQ3. However, when exposed to MMP-2, the quenched fluorescence was recovered by peptide cleavage. Following intravenous injection of the targeted AuNPs in mice bearing MMP-2-positive, HT-29 tumors, the targeted nanoparticles selectively accumulated in tumors, as observed by CT/fluorescence imaging (Sun et al., 2011). Deng et al. developed molecular beacons based on rod-shaped AuNPs for the targeted *in vivo* detection of matriptase expression on tumors. The molecular beacons consisted of an activatable fluorophore-peptide conjugate attached to AuNPs. The quenched fluorescence of the fluorophore by the AuNPs was restored when exposed to matriptase. After intratumoral injection of the targeted AuNPs in nude mice bearing HT-29 tumor xenografts with high matriptase expression, a strong fluorescence signal was observed in the tumors after 8 h, clearly indicating the tumor-targeting ability of the AuNPs (Deng et al., 2013). Multifunctional nanoparticles based on AuNRs were developed for targeted positron emission tomography (PET) imaging of and therapy for cancer. The multifunctional nanoparticle system consisted of PEGylated AuNRs; cyclic RGD and a ^64^Cu-chelator were attached to the distal ends of PEG, and DOX was conjugated to the AuNRs via a linker that was vulnerable to tumor acidity. The AuNRs demonstrated target specificity in a relevant model both *in vitro* and *in vivo*. However, in U87MG tumor-bearing mice, PET imaging revealed similar biodistribution of both targeted and non-targeted AuNRs (Xiao et al., 2012). Lee et al. used heparinase-specific targeted AuNPs (AuNP-HHep)-based activatable probes to detect and induce apoptosis in metastatic cancer cells. AuNP-HHep consisted of AuNPs conjugated to fluorophore-labeled heparin and RGD-bearing PEG. The fluorescence was quenched by the surface of the AuNPs and was recovered after exposure to the enzyme heparinase, which is known to be overexpressed in metastatic cancer cells. In addition, this targeted probe demonstrated highly specific apoptotic activity for cancer cells over-expressing α_*v*_β_3_ on their membranes, revealing that the accumulation of heparin within the cells triggered apoptotic events. The probe employed in this study could be useful for both imaging and treatment of metastatic cancers (Lee et al., 2010b).

#### Clearance of AuNPs

AuNPs can be excreted by kidney or liver, however, substantial research efforts are needed to determine the factors that affect mode of excretion. Balasubramanian et al. investigated the biodistribution of AuNPs and found that 20 nm particles accumulated in the kidneys starting at 1 month with a decrease in Au levels in urine. It was explained that AuNPs may adsorb to the serum proteins or aggregate over time, which increased the particle size making them difficult to pass through glomerular membrane. Authors suggested that prolonged accumulation of AuNPs in the kidneys could damage the glomerular membrane and reduce the excretion (Balasubramanian et al., 2010). Zhang et al. investigated the biodistribution of two PEGylated AuNPs with sizes 20 and 80 nm in diameter. Authors found that size of NPs strongly influence the biodistribution. For instance, 20 nm AuNPs demonstrated prolonged blood circulation and reduced uptake by the liver and the spleen compared to 80-nm AuNPs. Importantly, 20-nm AuNPs also excreted from the body, suggesting the validity of AuNPs with size less than 20 nm in diameter for *in vivo* applications (Zhang et al., 2009).

In their work, Lipka et al. showed that PEG (10 kDa) modified AuNPs with a HD of 31 nm showed the most clearance via the hepato-biliary pathway compared to PEG (750 Da) modified AuNPs with a HD of 21 nm. In this study, AuNPs with PEG (10 kDa) are the NPs with the greatest diameter and a study by Johnston et al. (2010) showed that 1.4 nm NPs exhibited higher hepato-biliary clearance compared to 18 nm. From these results, authors suggested that bile excretion may be significantly dependent on particles PEGylation but not on their size (Lipka et al., 2010).

## Polymeric nanoparticles

In addition to metallic nanoparticles, the use of polymeric nanoparticles (PN) as imaging probes have received significant attention due to their unique properties. PN-based imaging probes have several advantages over conventional low-molecular-weight probes, including prolonged circulation time, improved stability, and target specificity, passivity to the immune system and little or no toxicity. In recent years, biological science in association with polymer science has yielded novel hybrid materials with sophisticated architectures for clinical imaging and therapy (Duncan, 2003; Mitra et al., 2005; Lee et al., 2010a; Park, 2012).

Scherer et al. developed NIR polymer-based proteolytic beacons (PB-M7NIR) for the *in vivo* detection and quantitation of MMP-7 activity. PB-M7NIR was a PEGylated PAMAM-G4 dendrimer that was covalently coupled to a Cy5.5-labeled peptide substrate [Cy5.5-ahx-RPLALWRS(ahx)C] that monitored MMP-7 activity and to a quencher (AF750). *In vitro* OI studies showed a five-fold increase in fluorescence. The intravenous administration of PB-M7NIR in mice allowed for selective tumor visualization and localization with two point two-fold improved signals in MMP7 SW480 tumors relative to MMP7-negative tumors (Scherer et al., 2008). Oslon et al. developed activatable cell-penetrating peptide (ACCP)-conjugated bimodal G5 PAMAM dendrimer nanoparticles (ACCPDs) labeled with Cy5, Gd, or both. Cellular uptake of these nanoparticles in tumors was four to fifteen-fold higher than that of bare ACPPs. With OI, residual tumors and metastases as small as 200 μm were detected. ACCPDs were also used to visualize tumors during surgery (Olson et al., 2009). Successful surgical removal of tumors depends on the surgeon's ability to recognize and differentiate tumors from normal tissues; thus, it would be beneficial to objectively assess tumor margins while performing surgery. ACCPDs demonstrated superior ability to identify tumor tissue in tumor-bearing mice during surgery relative to ACCPs. As a result, fewer residual cancer cells were left in the animals following surgery, and the animals showed prolonged tumor-free survival and overall survival relative to animals resected using traditional bright-field illumination (Nguyen et al., 2010). In another study, Galande et al. developed self-quenched, dendrimeric peptide-based probes using multiple antigenic peptide (MAP), which fluoresced only after enzymatic treatment. The peptide-based probe was developed on a solid support that consisted of a dipeptide cathepsin S substrate (Leu-Arg) and PEG chains on the dendritic arms. An NIR dye, CyTE777, was attached to the N-terminus of the peptide to establish a dye-dye self-quenching system. *In vitro* studies showed that in the presence of protease cathepsin S, the peptide-based probe demonstrated a more than seventy-fold increase in and more than 95% recovery of fluorescence emission (Galande et al., 2006).

**Figure 7:**
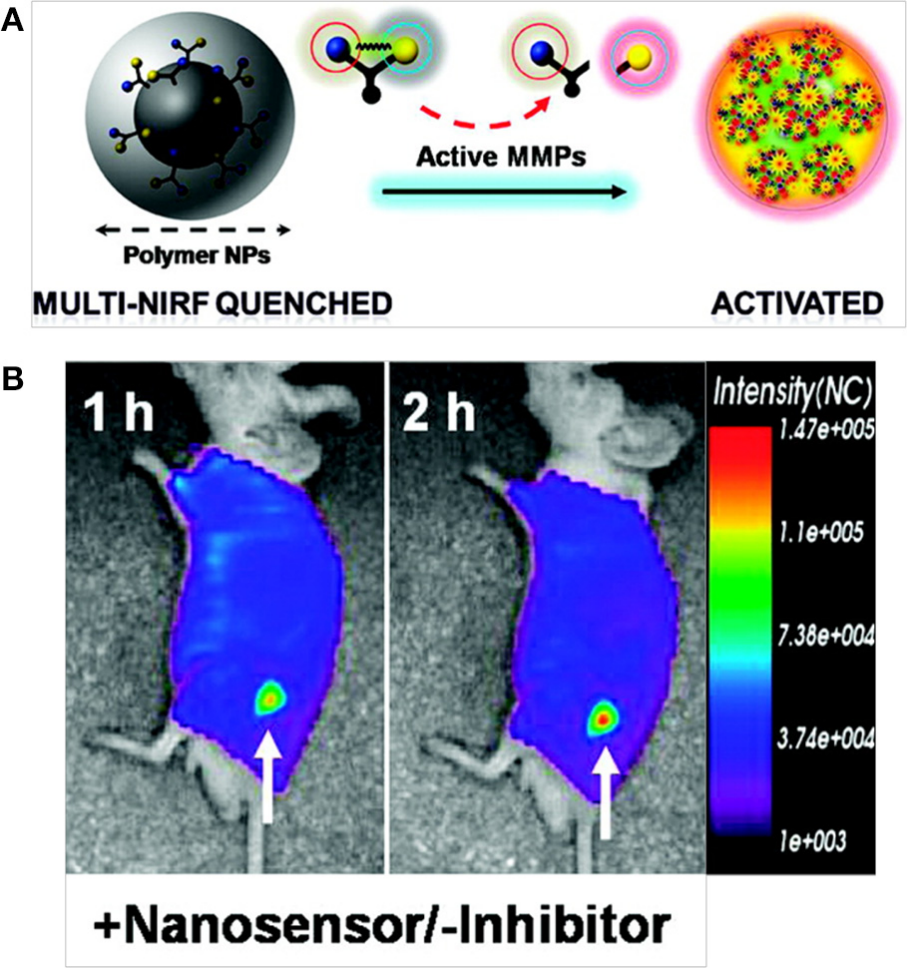
**(A)** MMP-sensitive NS and **(B)** *in vivo* NIR fluorescence tomographic images of subcutaneous SCC7 tumor-bearing mice after intravenous injection of the NS (Lee et al., 2009), © 2009, American Chemical Society.

Lee et al. developed a self-assembled, chitosan nanoparticle-based nanosensor (NS) that consisted of activatable MMP-specific NIR fluorogenic peptides with an NIR dark quencher BHQ-3 (Figure 7). This type of peptide substrate-mediated fluorescence labeling of nanoparticles could be strongly dual-quenched by both the dye-dark quencher and the NIR dye-dye self-quenching mechanisms. In the presence of MMP, the NIR-dye substrate was cleaved, which resulted in NIR fluorescence signal recovery. Intravenously administered NSs demonstrated enzyme specificity in both MMP-positive, SCC-7 xenograft tumor and colon cancer mouse models (Lee et al., 2009). Kim et al. developed a polymeric nanoparticle-based probe that could sense early apoptosis. The probe was an on/off system that consisted of a caspase-3 cleavable, NIR dye peptide substrate (Cy5.5-DEVDC) covalently attached to a deoxycholic acid-bearing polyethyleneamine conjugate. Due to the close proximity of Cy5.5 dyes to the nanoparticle surface, the fluorescence was self-quenched (off state). In the presence of caspase-3 enzyme *in vitro*, the nanoparticle recovered fluorescence (a ten-fold increase) that allowed monitoring of caspase-dependent apoptosis induced by tumor necrosis factor apoptosis inducing ligand in living HeLa cells (Kim et al., 2006).

### Clearance of PN

Gref et al. studied the effect of PEG chain length (2 and 5 kDa) in preventing plasma protein adsorption on the surface of poly(lactic acid)-PEG (PLA-PEG) NPs. PLA-PEG (5 kDa) demonstrated minimal protein adsorption (~80%) compared to non-PEGylated PLA NPs. PEG content between 2 and 5% was determined as a threshold value for optimal protein resistance. Interestingly, distances of 1–2 nm between two terminally attached PEG chains were estimated for minimal protein adsorption (Gref et al., 2000). Using polystyrene NPs of two sizes 50 and 500 nm, Kimura et al. studied the relation between protein adsorption kinetics and their liver uptake. 50 nm NPs exhibited rapid hepatic uptake when incubated with serum before transfusion in the rat liver. *In vivo* biodistribution of particle sizes of 50 and 500 nm revealed significant levels of agglomeration of larger particles in the liver. The surface charge of NPs has profound effect on uptake by the phagocytic system. Positively charged NPs are expected to have high non-specific internalization rate and short half-life compared to neutral or negatively charged NPs (Nagayama et al., 2007).

Kataoka et al. investigated the biodistribution of tyrosine (tyr) and tyrosine-glutamic acid (tyr-glu) conjugated PEG-poly (_D,L_-lactide) micelles in mice. Both of these micelles [tyr (neutral) and tyr-glu (negative)] showed a significant long circulation time in the blood compartment with 25% of injected dose still circulating even at 24 h. Biodistribution studies revealed that anionic micelle exhibited lower uptake into the liver and spleen compared to a neutral tyr-conjugated micelle, suggesting a substantial role of the anionic charge in avoiding non-specific organ uptake (Yamamoto et al., 2001).

## Conclusion and perspectives

The current medical imaging technologies used in the clinic for cancer diagnosis and staging depend on anatomical changes. The advent of MI allows expression identification of specific biomolecules based on functional changes that are correlated with the genesis of cancer, which greatly enhances our understanding of disease mechanisms and progression. In addition, critical data obtained from MI would enable clinicians to plan and design treatments, which is crucial for patient compliance and survival. The development of targeted probes and optimizing their ability to reach a site of interest remains a herculean task. The inability of probes to cross various biological barriers largely accounts for their shortcomings. An ideal probe should essentially possess a high level of specificity for a target and should successfully overcome biological barriers.

Substantial developments in nanotechnology have resulted in the development of imaging probes functionalized with a diverse range of ligands. Due to the versatile nature of nanoparticles, creating custom-made probes with unique properties, such as superior targeting ability and enhanced *in vivo* half-life, is possible. Owing to these features, several multimodal imaging systems have been developed as powerful tools for accurate cancer diagnosis because they integrate more than one imaging technique. For instance, an MI probe that included NIR dye and MR contrast agents was developed and subsequently used for preoperative MRI and intraoperative brain tumor delineation through OI. Although nanoparticles have shown promising results as MI probes, proper safety evaluation regarding their toxicity is absolutely necessary before they enter clinical use.

### Conflict of interest statement

The authors declare that the research was conducted in the absence of any commercial or financial relationships that could be construed as a potential conflict of interest.

## References

[B1] Åkerman M. E. Chan W. C. W. Laakkonen P. Bhatia S. N. Ruoslahti E. (2002). Nanocrystal targeting *in vivo*. Proc. Natl. Acad. Sci. U.S.A. 99, 12617–12621. 10.1073/pnas.152463399 12235356 PMC130509

[B2] Antony A. C. (1996). Folate receptors. Ann. Rev. Nutr. 16, 501–521. 10.1146/annurev.nu.16.070196.002441 8839936

[B3] Anzai Y. Piccoli C. W. Outwater E. K. Stanford W. Bluemke D. A. Nurenberg P.. (2003). Evaluation of neck and body metastases to nodes with Ferumoxtran 10–enhanced MR imaging: phase III safety and efficacy study. Radiology 228, 777–788. 10.1148/radiol.2283020872 12954896

[B4] Bagalkot V. Zhang L. Levy-Nissenbaum E. Jon S. Kantoff P. W. Langer R.. (2007). Quantum Dot-Aptamer conjugates for synchronous cancer imaging, therapy, and sensing of drug delivery based on bi-fluorescence resonance energy transfer. Nano Lett. 7, 3065–3070. 10.1021/nl071546n 17854227

[B5] Balasubramanian S. K. Jittiwat J. Manikandan J. Ong C. N. Yu L. E. Ong W. Y. (2010). Biodistribution of gold nanoparticles and gene expression changes in the liver and spleen after intravenous administration in rats. Biomaterials 31, 2034–2042. 10.1016/j.biomaterials.2009.11.079 20044133

[B6] Ballou B. Fisher G. W. Waggoner A. S. Farkas D. L. Reiland J. M. Jaffe R.. (1995). Tumor labeling *in vivo* using cyanine-conjugated monoclonal antibodies. Cancer Immunol. Immunother. 41, 257–263. 10.1007/BF01517001 7489569 PMC11037679

[B7] Becker A. Hessenius C. Licha K. Ebert B. Sukowski U. Semmler W.. (2001). Receptor-targeted optical imaging of tumors with near-infrared fluorescent ligands. Nat. Biotechnol. 19, 327–331. 10.1038/86707 11283589

[B8] Boisselier E. Astruc D. (2009). Gold nanoparticles in nanomedicine: preparations, imaging, diagnostics, therapies and toxicity. Chem. Soc. Rev. 38, 1759–1782. 10.1039/b806051g 19587967

[B9] Bourrinet P. Bengele H. H. Bonnemain B. Dencausse A. Idee J. M. Jacobs P. M.. (2006). Preclinical safety and pharmacokinetic profile of ferumoxtran-10, an ultrasmall superparamagnetic iron oxide magnetic resonance contrast agent. Invest. Radiol. 41, 313–324. 10.1097/01.rli.0000197669.80475.dd 16481915

[B10] Bruchez M. Moronne M. Gin P. Weiss S. Alivisatos A. P. (1998). Semiconductor nanocrystals as fluorescent biological labels. Science 281, 2013–2016. 10.1126/science.281.5385.2013 9748157

[B11] Cai W. Shin D. W. Chen K. Gheysens O. Cao Q. Wang S. X.. (2006). Peptide-labeled near-infrared quantum dots for imaging tumor vasculature in living subjects. Nano Lett. 6, 669–676. 10.1021/nl052405t 16608262

[B12] Caravan P. Ellison J. J. McMurry T. J. Lauffer R. B. (1999). Gadolinium(III) chelates as MRI contrast agents: structure, dynamics, and applications. Chem. Rev. 99, 2293–2352. 10.1021/cr980440x 11749483

[B13] Chanda N. Kattumuri V. Shukla R. Zambre A. Katti K. Upendran A.. (2010). Bombesin functionalized gold nanoparticles show *in vitro* and *in vivo* cancer receptor specificity. Proc. Natl. Acad. Sci. U.S.A. 107, 8760–8765. 10.1073/pnas.1002143107 20410458 PMC2889350

[B14] Chang E. Miller J. S. Sun J. Yu W. W. Colvin V. L. Drezek R.. (2005). Protease-activated quantum dot probes. Biochem. Biophy. Res. Commun 334, 1317–1321. 10.1016/j.bbrc.2005.07.028 16039606

[B15] Chen Q. Li K. Wen S. Liu H. Peng C. Cai H.. (2013). Targeted CT/MR dual mode imaging of tumors using multifunctional dendrimer-entrapped gold nanoparticles. Biomaterials 34, 5200–5209. 10.1016/j.biomaterials.2013.03.009 23583039

[B16] Chen T. J. Cheng T. H. Chen C. Y. Hsu S. C. Cheng T. L. Liu G. C.. (2009). Targeted Herceptin–dextran iron oxide nanoparticles for noninvasive imaging of HER2/neu receptors using MRI. J. Biol. Inorg. Chem. 14, 253–260. 10.1007/s00775-008-0445-9 18975017

[B17] Cheng K. Cheng Z. (2012). Near infrared receptor-targeted nanoprobes for early diagnosis of cancers. Curr. Med. Chem. 19, 4767–4785. 10.2174/092986712803341458 22873665

[B18] Cheng W. Ping Y. Zhang Y. Chuang K.-H. Liu Y. (2013). Magnetic resonance imaging (MRI) contrast agents for tumor diagnosis. J. Healthc. Eng. 4, 23–46. 10.1260/2040-2295.4.1.23 23502248

[B19] Cheng Z. Thorek D. L. J. Tsourkas A. (2010). Gadolinium-conjugated dendrimer nanoclusters as a tumor-targeted T1 magnetic resonance imaging contrast agent. Angew. Chem. Int. Ed. 49, 346–350. 10.1002/anie.200905133 19967688 PMC2862691

[B20] Choi H. Choi S. R. Zhou R. Kung H. F. Chen I. W. (2004). Iron oxide nanoparticles as magnetic resonance contrast agent for tumor imaging via folate receptor-targeted delivery1. Acad. Radiol. 11, 996–1004. 10.1016/j.acra.2004.04.018 15350580

[B21] Choi H. S. Liu W. Misra P. Tanaka E. Zimmer J. P. Ipe B. I.. (2007). Renal clearance of quantum dots. Nat. Biotechnol. 25, 1165–1170. 10.1038/nbt1340 17891134 PMC2702539

[B22] Choi Y.-E. Kwak J.-W. Park J. W. (2010). Nanotechnology for early cancer detection. Sensors 10, 428–455. 10.3390/s100100428 22315549 PMC3270850

[B23] Corot C. Robert P. Idée J.-M. Port M. (2006). Recent advances in iron oxide nanocrystal technology for medical imaging. Adv. Drug Deliv. Rev. 58, 1471–1504. 10.1016/j.addr.2006.09.013 17116343

[B24] Couvreur P. Vauthier C. (2006). Nanotechnology: intelligent design to treat complex disease. Pharm. Res. 23, 1417–1450. 10.1007/s11095-006-0284-8 16779701

[B25] Damadian R. (1971). Tumor detection by nuclear magnetic resonance. Science 171, 1151–1153. 10.1126/science.171.3976.1151 5544870

[B26] Deng D. Zhang D. Li Y. Achilefu S. Gu Y. (2013). Gold nanoparticles based molecular beacons for *in vitro* and *in vivo* detection of the matriptase expression on tumor. Biosens. Bioelectron. 49, 216–221. 10.1016/j.bios.2013.05.018 23770391

[B27] Diagaradjane P. Orenstein-Cardona J. M. Colón-Casasnovas N. E. Deorukhkar A. Shentu S. Kuno N.. (2008). Imaging epidermal growth factor receptor expression *in vivo*: pharmacokinetic and biodistribution characterization of a bioconjugated quantum dot nanoprobe. Clin. Cancer Res. 14, 731–741. 10.1158/1078-0432.CCR-07-1958 18245533

[B28] Duncan R. (2003). The dawning era of polymer therapeutics. Nat. Rev. Drug Discov. 2, 347–360. 10.1038/nrd1088 12750738

[B29] Enochs W. S. Harsh G. Hochberg F. Weissleder R. (1999). Improved delineation of human brain tumors on MR images using a long-circulating, superparamagnetic iron oxide agent. J. Magn. Reson. Imaging 9, 228–232. 10.1002/(SICI)1522-2586(199902)9:2<228::AID-JMRI12>3.0.CO;2-K 10077018

[B30] Fass L. (2008). Imaging and cancer: a review. Mol. Oncol. 2, 115–152. 10.1016/j.molonc.2008.04.001 19383333 PMC5527766

[B31] Ferrari M. (2005). Cancer nanotechnology: opportunities and challenges. Nat. Rev. Cancer 5, 161–171. 10.1038/nrc1566 15738981

[B32] Fischer H. C. Liu L. Pang K. S. Chan W. C. W. (2006). Pharmacokinetics of nanoscale quantum dots: *in vivo* distribution, sequestration, and clearance in the rat. Adv. Funct. Mater. 16, 1299–1305 10.1002/adfm.200500529

[B33] Galande A. Hilderbrand S. Weissleder R. Tung C. (2006). Enzyme-targeted fluorescent imaging probes on a multiple antigenic peptide core. J. Med. Chem. 49, 4715–4720. 10.1021/jm051001a 16854078

[B34] Gao J. Chen K. Miao Z. Ren G. Chen X. Gambhir S. S.. (2011). Affibody-based nanoprobes for HER2-expressing cell and tumor imaging. Biomaterials 32, 2141–2148. 10.1016/j.biomaterials.2010.11.053 21147502 PMC3032351

[B35] Gao X. Cui Y. Levenson R. M. Chung L. W. K. Nie S. (2004). *In vivo* cancer targeting and imaging with semiconductor quantum dots. Nat. Biotechnol. 22, 969–976. 10.1038/nbt994 15258594

[B36] Gref R. Lück M. Quellec P. Marchand M. Dellacherie E. Harnisch S.. (2000). ‘Stealth’ corona-core nanoparticles surface modified by polyethylene glycol (PEG): influences of the corona (PEG chain length and surface density) and of the core composition on phagocytic uptake and plasma protein adsorption. Colloids Surf. B 18, 301–313. 10.1016/S0927-7765(99)00156-3 10915952

[B37] Han B. Nakamura M. Mori I. Nakamura Y. Kakudo K. (2005). Urokinase-type plasminogen activator system and breast cancer. Oncol. Rep. 14, 105–112. 10.3892/or.14.1.105 15944776

[B38] Han L. Huang R. Li J. Liu S. Huang S. Jiang C. (2011a). Plasmid pORF-hTRAIL and doxorubicin co-delivery targeting to tumor using peptide-conjugated polyamidoamine dendrimer. Biomaterials 32, 1242–1252. 10.1016/j.biomaterials.2010.09.070 20971503

[B39] Han L. Huang R. Liu S. Huang S. Jiang C. (2010). Peptide-conjugated PAMAM for targeted doxorubicin delivery to transferrin receptor overexpressed tumors. Mol. Pharm. 7, 2156–2165. 10.1021/mp100185f 20857964

[B40] Han L. Li J. Huang S. Huang R. Liu S. Hu X.. (2011b). Peptide-conjugated polyamidoamine dendrimer as a nanoscale tumor-targeted T1 magnetic resonance imaging contrast agent. Biomaterials 32, 2989–2998. 10.1016/j.biomaterials.2011.01.005 21277017

[B41] Herbst R. S. (2004). Review of epidermal growth factor receptor biology. Int. J. Radiat. Oncol. Biol. Phys. 59, S21–S26. 10.1016/j.ijrobp.2003.11.041 15142631

[B42] Hopkins C. R. Trowbridge I. S. (1983). Internalization and processing of transferrin and the transferrin receptor in human carcinoma A431 cells. J. Cell Biol. 97, 508–521. 10.1083/jcb.97.2.508 6309862 PMC2112524

[B43] Huang R. Han L. Li J. Liu S. Shao K. Kuang Y.. (2011). Chlorotoxin-modified macromolecular contrast agent for MRI tumor diagnosis. Biomaterials 32, 5177–5186. 10.1016/j.biomaterials.2011.03.075 21531455

[B44] Hussain T. Nguyen Q. T. (2014). Molecular imaging for cancer diagnosis and surgery. Adv. Drug Deliv. Rev. 66, 90–100. 10.1016/j.addr.2013.09.007 24064465 PMC4464660

[B45] Islam T. Josephson L. (2009). Current state and future applications of active targeting in malignancies using superparamagnetic iron oxide nanoparticles. Cance Biomark 5, 99–107. 10.3233/CBM-2009-0615 19414927 PMC12922813

[B46] Jacobs R. E. Cherry S. R. (2001). Complementary emerging techniques: high-resolution PET and MRI. Curr. Opin. Neurobiol. 11, 621–629. 10.1016/S0959-4388(00)00259-2 11595498

[B47] Jaffer F. A. Weissleder R. (2005). MOlecular imaging in the clinical arena. JAMA 293, 855–862. 10.1001/jama.293.7.855 15713776

[B48] Jain T. K. Reddy M. K. Morales M. A. Leslie-Pelecky D. L. Labhasetwar V. (2008). Biodistribution, clearance, and biocompatibility of iron oxide magnetic nanoparticles in rats. Mol. Pharm. 5, 316–327. 10.1021/mp7001285 18217714

[B49] Jemal A. Bray F. Center M. M. Ferlay J. Ward E. Forman D. (2011). Global cancer statistics. CA Cancer J. Clin. 61, 69–90. 10.3322/caac.20107 21296855

[B50] Johnston H. J. Semmler-Behnke M. Brown D. M. Kreyling W. Tran L. Stone V. (2010). Evaluating the uptake and intracellular fate of polystyrene nanoparticles by primary and hepatocyte cell lines *in vitro*. Toxicol. Appl. Pharmacol. 242, 66–78. 10.1016/j.taap.2009.09.015 19799923

[B51] Juweid M. E. Cheson B. D. (2006). Positron-emission tomography and assessment of cancer therapy. New Engl. J. Med. 354, 496–507. 10.1056/NEJMra050276 16452561

[B52] Kelly K. A. Setlur S. R. Ross R. Anbazhagan R. Waterman P. Rubin M. A.. (2008). Detection of early prostate cancer using a hepsin-targeted imaging agent. Cancer Res. 68, 2286–2291. 10.1158/0008-5472.CAN-07-1349 18381435 PMC2709884

[B53] Kim D. Jeong Y. Y. Jon S. (2010). A drug-loaded Aptamer-Gold nanoparticle bioconjugate for combined CT imaging and therapy of prostate cancer. ACS Nano 4, 3689–3696. 10.1021/nn901877h 20550178

[B54] Kim K. Lee M. Park H. Kim J. H. Kim S. Chung H.. (2006). Cell-Permeable and Biocompatible Polymeric Nanoparticles for Apoptosis Imaging. J. Am. Chem. Soc. 128, 3490–3491. 10.1021/ja057712f 16536501

[B55] Kobayashi H. Brechbiel M. (2005). Nano-sized MRI contrast agents with dendrimer cores. Adv. Drug Deliv. Rev. 57, 2271–2286. 10.1016/j.addr.2005.09.016 16290152

[B56] Kobayashi H. Brechbiel M. W. (2003). Dendrimer-based macromolecular MRI contrast agents: characteristics and application. Mol. Imaging 2, 1–10. 10.1162/153535003765276237 12926232

[B57] Kresse M. Wagner S. Pfefferer D. Lawaczeck R. Elste V. Semmler W. (1998). Targeting of ultrasmall superparamagnetic iron oxide (USPIO) particles to tumor cells *in vivo* by using transferrin receptor pathways. Magn. Reson. Med. 40, 236–242. 10.1002/mrm.1910400209 9702705

[B58] Kumar A. Zhang X. Liang X.-J. (2013). Gold nanoparticles: emerging paradigm for targeted drug delivery system. Biotechnol. Adv. 31, 593–606. 10.1016/j.biotechadv.2012.10.002 23111203

[B59] LaVan D. A. McGuire T. Langer R. (2003). Small-scale systems for *in vivo* drug delivery. Nat. Biotechnol. 21, 1184–1191. 10.1038/nbt876 14520404

[B60] Lee K. Lee H. Bae K. H. Park T. G. (2010b). Heparin immobilized gold nanoparticles for targeted detection and apoptotic death of metastatic cancer cells. Biomaterials 31, 6530–6536. 10.1016/j.biomaterials.2010.04.046 20537379

[B61] Lee S. Cha E. J. Park K. Lee S. Y. Hong J. K. Sun I. C.. (2008). A near-infrared-fluorescence-quenched gold-nanoparticle imaging probe for *in vivo* drug screening and protease activity determination. Angew. Chem. Int. Ed. 120, 2846–2849. 10.1002/ange.200705240 18306196

[B62] Lee S. H. Kim B. H. Na H. B. Hyeon T. (2014). Paramagnetic inorganic nanoparticles as T1MRI contrast agents. Wiley Interdiscip. Rev. Nanomed. Nanobiotechnol. 6, 196–209. 10.1002/wnan.1243 24123961

[B63] Lee S. Ryu J. H. Park K. Lee A. Lee S. Y. Youn I. C.. (2009). Polymeric nanoparticle-based activatable near-infrared nanosensor for protease determination *in vivo*. Nano Lett. 9, 4412–4416. 10.1021/nl902709m 19842672 PMC3618996

[B64] Lee S. Xie J. Chen X. (2010a). Activatable molecular probes for cancer imaging. Curr. Top. Med. Chem. 10, 1135–1144. 10.2174/156802610791384270 20388112 PMC3629980

[B65] Leuschner C. Kumar, Challa S. S. R. Hansel W. Hormes J. (2005). Targeting breast cancer cells and their metastases through luteinizing hormone releasing hormone (LHRH) receptors using magnetic nanoparticles. J. Biomed. Nanotechnol. 1, 229–233 10.1166/jbn.2005.027

[B66] Li X. Du X. Huo T. Liu X. Zhang S. Yuan F. (2009). Specific targeting of breast tumor by octreotide-conjugated ultrasmall superparamagnetic iron oxide particles using a clinical 3. 0-tesla magnetic resonance scanner. Acta. Radiol. 50, 583–594. 10.1080/02841850902902557 19449236

[B67] Lin W. Hyeon T. Lanza G. M. Zhang M. Meade T. J. (2009). Magnetic nanoparticles for early detection of cancer by magnetic resonance imaging. MRS Bull. 34, 441–448 10.1557/mrs2009.120 PMC4495966 26166945

[B68] Lipka J. Semmler-Behnke M. Sperling R. A. Wenk A. Takenaka S. Schleh C.. (2010). Biodistribution of PEG-modified gold nanoparticles following intratracheal instillation and intravenous injection. Biomaterials 31, 6574–6581. 10.1016/j.biomaterials.2010.05.009 20542560

[B69] Lu Y. Low P. S. (2002). Folate-mediated delivery of macromolecular anticancer therapeutic agents. Adv. Drug Deliv. Rev. 54, 675–693. 10.1016/S0169-409X(02)00042-X 12204598

[B70] Luker G. D. Luker K. E. (2008). Optical imaging: current applications and future directions. J. Nucl. Med. 49, 1–4. 10.2967/jnumed.107.045799 18077528

[B71] Luo K. Liu G. She W. Wang Q. Wang G. He B. (2011). Gadolinium-labeled peptide dendrimers with controlled structures as potential magnetic resonance imaging contrast agents. Biomaterials 32, 7951–7960 10.1016/j.biomaterials.2011.07.006 21784511

[B72] Martin S. J. Reutelingsperger C. P. McGahon A. J. Rader J. A. van Schie R. C. LaFace D. M. (1995). Early redistribution of plasma membrane phosphatidylserine is a general feature of apoptosis regardless of the initiating stimulus: inhibition by overexpression of Bcl-2 and Abl. J. Exp. Med. 182, 1545–1556 10.1084/jem.182.5.1545 7595224 PMC2192182

[B73] Massoud T. F. Gambhir S. S. (2003). Molecular imaging in living subjects: seeing fundamental biological processes in a new light. Gene. Dev. 17, 545–580. 10.1101/gad.1047403 12629038

[B74] McCarthy J. R. Bhaumik J. Karver M. R. Sibel Erdem S. Weissleder R. (2010). Targeted nanoagents for the detection of cancers. Mol. Oncol. 4, 511–528. 10.1016/j.molonc.2010.08.003 20851695 PMC2981649

[B75] McCarthy J. R. Weissleder R. (2008). Multifunctional magnetic nanoparticles for targeted imaging and therapy. Adv. Drug Deliv. Rev. 60, 1241–1251. 10.1016/j.addr.2008.03.014 18508157 PMC2583936

[B76] Medintz I. L. Uyeda H. T. Goldman E. R. Mattoussi H. (2005). Quantum dot bioconjugates for imaging, labelling and sensing. Nat. Mater. 4, 435–446. 10.1038/nmat1390 15928695

[B77] Meng J. Fan J. Galiana G. Branca R. T. Clasen P. L. Mad S. (2009). LHRH-functionalized superparamagnetic iron oxide nanoparticles for breast cancer targeting and contrast enhancement in MRI. Mater. Sci. Eng. C 29, 1467–1479 10.1016/j.msec.2008.09.039

[B78] Meng X. X. Wan J. Q. Jing M. Zhao S. G. Cai W. Liu E. Z. (2007). Specific targeting of gliomas with multifunctional superparamagnetic iron oxide nanoparticle optical and magnetic resonance imaging contrast agents1. Acta Pharmacol. Sin. 28, 2019–2026 10.1111/j.1745-7254.2007.00661.x 18031618

[B79] Michalet X. Pinaud F. F. Bentolila L. A. Tsay J. M. Doose S. Li J. J.. (2005). Quantum dots for live cells, *in vivo* imaging, and diagnostics. Science 307, 538–544. 10.1126/science.1104274 15681376 PMC1201471

[B80] Mitra A. Mulholland J. Nan A. McNeill E. Ghandehari H. Line B. R. (2005). Targeting tumor angiogenic vasculature using polymer–RGD conjugates. J. Control. Release 102, 191–201. 10.1016/j.jconrel.2004.09.023 15653145

[B81] Morgan S. Grootendorst P. Lexchin J. Cunningham C. Greyson D. (2011). The cost of drug development: a systematic review. Health Policy 100, 4–17. 10.1016/j.healthpol.2010.12.002 21256615

[B82] Mu C. J. LaVan D. A. Langer R. S. Zetter B. R. (2010). Self-assembled gold nanoparticle molecular probes for detecting proteolytic activity *in vivo*. ACS Nano 4, 1511–1520. 10.1021/nn9017334 20146506 PMC2847389

[B83] Mulder W. J. Koole R. Brandwijk R. J. Storm G. Chin P. T. Strijkers G. J. (2005). Quantum dots with a paramagnetic coating as a bimodal molecular imaging probe. Nano Lett. 6, 1–6 10.1021/nl051935m 16402777

[B84] Nagayama S. Ogawara K.-I. Fukuoka Y. Higaki K. Kimura T. (2007). Time-dependent changes in opsonin amount associated on nanoparticles alter their hepatic uptake characteristics. Int. J. Pharm. 342, 215–221 10.1016/j.ijpharm.2007.04.036 17566676

[B85] Neuwelt E. A. Várallyay P. Bagó A. G. Muldoon L. L. Nesbit G. Nixon R. (2004). Imaging of iron oxide nanoparticles by MR and light microscopy in patients with malignant brain tumours. Neuropathol. Appl. Neurobiol. 30, 456–471. 10.1111/j.1365-2990.2004.00557.x 15488022

[B86] Nguyen Q. Olson E. S. Aguilera T. A. Jiang T. Scadeng M. Ellies L. G.. (2010). Surgery with molecular fluorescence imaging using activatable cell-penetrating peptides decreases residual cancer and improves survival. Proc. Natl. Acad. Sci. U.S.A. 107, 4317–4322. 10.1073/pnas.0910261107 20160097 PMC2840114

[B87] Olson E. S. Aguilera T. A. Jiang T. Ellies L. G. Nguyen Q. T. Wong E. H.. (2009). *In vivo* characterization of activatable cell penetrating peptides for targeting protease activity in cancer. Integr. Biol. 1, 382–393. 10.1039/b904890a 20023745 PMC2796841

[B88] Olson E. S. Jiang T. Aguilera T. A. Nguyen Q. T. Ellies L. G. Scadeng M.. (2010). Activatable cell penetrating peptides linked to nanoparticles as dual probes for *in vivo* fluorescence and MR imaging of proteases. Proc. Natl. Acad. Sci. U.S.A. 107, 4311–4316. 10.1073/pnas.0910283107 20160077 PMC2840175

[B89] Park K. (2012). Polysaccharide-based near-infrared fluorescence nanoprobes for cancer diagnosis. Quant. Imaging Med. Surg. 2, 106–113. 10.3978/j.issn.2223-4292.2012.05.01 23256067 PMC3508597

[B90] Peer D. Karp J. M. Hong S. Farokhzad O. C. Margalit R. Langer R. (2007). Nanocarriers as an emerging platform for cancer therapy. Nat. Nanotechnol. 2, 751–760. 10.1038/nnano.2007.387 18654426

[B91] Pericleous P. Gazouli M. Lyberopoulou A. Rizos S. Nikiteas N. Efstathopoulos E. P. (2012). Quantum dots hold promise for early cancer imaging and detection. Int. J. Cancer 131, 519–528. 10.1002/ijc.27528 22411309

[B92] Pham W. Choi Y. Weissleder R. Tung C.-H. (2004). Developing a Peptide-Based Near-Infrared Molecular Probe for Protease Sensing. Bioconjug. Chem 15, 1403–1407. 10.1021/bc049924s 15546208

[B93] Pilch J. Brown D. M. Komatsu M. Järvinen T. A. Yang M. Peters D.. (2006). Peptides selected for binding to clotted plasma accumulate in tumor stroma and wounds. Proc. Natl. Acad. Sci. U.S.A. 103, 2800–2804. 10.1073/pnas.0511219103 16476999 PMC1413849

[B94] Popovtzer R. Agrawal A. Kotov N. A. Popovtzer A. Balter J. Carey T. E.. (2008). Targeted gold nanoparticles enable molecular CT imaging of cancer. Nano Lett. 8, 4593–4596. 10.1021/nl8029114 19367807 PMC2772154

[B95] Pysz M. A. Gambhir S. S. Willmann J. K. (2010). Molecular imaging: current status and emerging strategies. Clin. Radiol. 65, 500–516. 10.1016/j.crad.2010.03.011 20541650 PMC3150531

[B96] Qian J. Yong K. T. Roy I. Ohulchanskyy T. Y. Bergey E. J. Lee H. H.. (2007). Imaging pancreatic cancer using surface-functionalized quantum dots. J. Phys. Chem. B 111, 6969–6972. 10.1021/jp070620n 17552555

[B97] Regino C. A. Walbridge S. Bernardo M. Wong K. J. Johnson D. Lonser R.. (2008). A dual CT-MR dendrimer contrast agent as a surrogate marker for convection-enhanced delivery of intracerebral macromolecular therapeutic agents. Contrast Media Mol. Imaging 3, 2–8. 10.1002/cmmi.223 18335478 PMC2278235

[B98] Rudin M. Weissleder R. (2003). Molecular imaging in drug discovery and development. Nat. Rev. Drug Discov. 2, 123–131. 10.1038/nrd1007 12563303

[B99] Saini S. Stark D. D. Hahn P. F. Wittenberg J. Brady T. J. Ferrucci J. T. Jr. (1987). Ferrite particles: a superparamagnetic MR contrast agent for the reticuloendothelial system. Radiology 162, 211–216. 10.1148/radiology.162.1.3786765 3786765

[B100] Saravanakumar G. Kim K. Park J. H. Rhee K. Kwon I. C. (2009). Current status of nanoparticle-based imaging agents for early diagnosis of cancer and atherosclerosis. J. Biomed. Nanotechnol. 5, 20–35. 10.1166/jbn.2009.030 20055103

[B101] Sarin H. Kanevsky A. S. Wu H. Brimacombe K. R. Fung S. H. Sousa A. A.. (2008). Effective transvascular delivery of nanoparticles across the blood-brain tumor barrier into malignant glioma cells. J. Transl. Med. 6:80. 10.1186/1479-5876-6-80 19094226 PMC2639552

[B102] Schellenberger E. A. Bogdanov A. Jr. Högemann D. Tait J. Weissleder R. Josephson L. (2002). Annexin V-CLIO: a nanoparticle for detecting apoptosis by MRI. Mol. Imaging 1, 102–107. 10.1162/153535002320162769 12920851

[B103] Scherer R. L. VanSaun M. N. McIntyre J. O. Matrisian L. M. (2008). Optical imaging of matrix metalloproteinase-7 activity *in vivo* using a proteolytic nanobeacon. Mol. Imaging 7, 118–1131. 10.2310/7290.2008.00010 19123982 PMC2777890

[B104] Schipper M. L. Cheng Z. Lee S. W. Bentolila L. A. Iyer G. Rao J.. (2007). microPET-based biodistribution of quantum dots in living mice. J. Nucl. Med. 48, 1511–1518. 10.2967/jnumed.107.040071 17704240 PMC4146342

[B105] Schnell O. Krebs B. Carlsen J. Miederer I. Goetz C. Goldbrunner R. H.. (2009). Imaging of integrin αvβ 3 expression in patients with malignant glioma by [18F] Galacto-RGD positron emission tomography. Neuro Oncol. 11, 861–870. 10.1215/15228517-2009-024 19401596 PMC2802406

[B106] Schroeder J. E. Shweky I. Shmeeda H. Banin U. Gabizon A. (2007). Folate-mediated tumor cell uptake of quantum dots entrapped in lipid nanoparticles. J. Control. Release 124, 28–34. 10.1016/j.jconrel.2007.08.028 17928088

[B107] Siegel R. Ma J. Zou Z. Jemal A. (2014). Cancer statistics, 2014. CA Cancer J. Clin. 64, 9–29. 10.3322/caac.21208 24399786

[B108] Smith B. R. Cheng Z. De A. Koh A. L. Sinclair R. Gambhir S. S. (2008). Real-time intravital imaging of RGD-quantum dot binding to luminal endothelium in mouse tumor neovasculature. Nano Lett. 8, 2599–2606. 10.1021/nl080141f 18386933 PMC4161135

[B109] Soroceanu L. Gillespie Y. Khazaeli M. B. Sontheimer H. (1998). Use of chlorotoxin for targeting of primary brain tumors. Cancer Res. 58, 4871–4879. 9809993

[B110] Sosnovik D. E. Weissleder R. (2007). Emerging concepts in molecular MRI. Curr. Opin. Biotechnol. 18, 4–10. 10.1016/j.copbio.2006.11.001 17126545

[B111] Stark D. D. Bradley W. J. Jr. (1999). Magnetic Resonance Imaging. St. Louis, MO: Mosby.

[B112] Sun C. Veiseh O. Gunn J. Fang C. Hansen S. Lee D.. (2008). *In vivo* MRI detection of gliomas by chlorotoxin-conjugated superparamagnetic nanoprobes. Small 4, 372–379. 10.1002/smll.200700784 18232053 PMC2692358

[B113] Sun I. C. Eun D. K. Koo H. Ko C. Y. Kim H. S. Yi D. K.. (2011). Tumor-targeting gold particles for dual computed tomography/optical cancer imaging. Angew. Chem. Int. Ed. 50, 9348–9351. 10.1002/anie.201102892 21948430

[B114] Swanson S. D. Kukowska-Latallo J. F. Patri A. K. Chen C. Ge S. Cao Z.. (2008). Targeted gadolinium-loaded dendrimer nanoparticles for tumor-specific magnetic resonance contrast enhancement. Int. J. Nanomedicine 3, 201–210. 10.2147/IJN.S2696 18686779 PMC2527674

[B115] Tan M. Ye Z. Lindner D. Brady-Kalnay S. Lu Z.-R. (2014). Synthesis and evaluation of a targeted nanoglobular dual-modal imaging agent for MR Imaging and image-guided surgery of prostate cancer. Pharm. Res. 31, 1–8. 10.1007/s11095-013-1008-5 23471641 PMC3895102

[B116] Thakor A. S. Jokerst J. Zavaleta C. Massoud T. F. Gambhir S. S. (2011). Gold nanoparticles: a revival in precious metal administration to patients. Nano Lett. 11, 4029–4036. 10.1021/nl202559p 21846107 PMC3195547

[B117] Tong L. Wei Q. Wei A. Cheng J.-X. (2009). Gold nanorods as contrast agents for biological imaging: optical properties, surface conjugation and photothermal effects. Photochem. Photobiol. 85, 21–32. 10.1111/j.1751-1097.2008.00507.x 19161395 PMC2818790

[B118] Tran T. Engfeldt T. Orlova A. Sandström M. Feldwisch J. Abrahmsén L.. (2007). 99mTc-maEEE-ZHER2:342, an affibody molecule-based tracer for the detection of HER2 expression in malignant tumors. Bioconjug. Chem. 18, 1956–1964. 10.1021/bc7002617 17944527

[B119] Weissleder R. (2002). Scaling down imaging: molecular mapping of cancer in mice. Nat. Rev. Cancer 2, 11–18. 10.1038/nrc701 11902581

[B120] Weissleder R. (2006). Molecular imaging in cancer. Science 312, 1168–1171. 10.1126/science.1125949 16728630

[B121] Weissleder R. Pittet M. J. (2008). Imaging in the era of molecular oncology. Nature 452, 580–589. 10.1038/nature06917 18385732 PMC2708079

[B122] Willmann J. K. van Bruggen N. Dinkelborg L. M. Gambhir S. S. (2008). Molecular imaging in drug development. Nat. Rev. Drug Discov. 7, 591–607. 10.1038/nrd2290 18591980

[B123] Xiao Y. Hong H. Matson V. Z. Javadi A. Xu W. Yang Y.. (2012). Gold Nanorods conjugated with doxorubicin and cRGD for combined anticancer drug delivery and PET imaging. Theranostics 2, 757–768. 10.7150/thno.4756 22916075 PMC3425121

[B124] Xu H. Regino C. A. Koyama Y. Hama Y. Gunn A. J. Bernardo M.. (2007). Preparation and preliminary evaluation of a biotin-targeted, lectin-targeted dendrimer-based probe for dual-modality magnetic resonance and fluorescence imaging. Bioconj. Chem 18, 1474–1482. 10.1021/bc0701085 17711320

[B125] Yamamoto Y. Nagasaki Y. Kato Y. Sugiyama Y. Kataoka K. (2001). Long-circulating poly(ethylene glycol)–poly(d,l-lactide) block copolymer micelles with modulated surface charge. J. Control. Release 77, 27–38. 10.1016/S0168-3659(01)00451-5 11689257

[B126] Yang L. Cao Z. Sajja H. K. Mao H. Wang L. Geng H.. (2008). Development of receptor targeted magnetic iron oxide nanoparticles for efficient drug delivery and tumor imaging. J. Biomed. Nanotechnol. 4, 439–449. 10.1166/jbn.2008.007 25152701 PMC4139059

[B127] Yang L. Mao H. Wang Y. A. Cao Z. Peng X. Wang X.. (2009b). Single chain epidermal growth factor receptor antibody conjugated nanoparticles for *in vivo* tumor targeting and imaging. Small 5, 235–243. 10.1002/smll.200800714 19089838 PMC3626261

[B128] Yang L. Peng X. H. Wang Y. A. Wang X. Cao Z. Ni C.. (2009a). Receptor-targeted nanoparticles for *in vivo* imaging of breast cancer. Clin. Cancer Res. 15, 4722–4732. 10.1158/1078-0432.CCR-08-3289 19584158 PMC2727672

[B129] Yong K. T. Ding H. Roy I. Law W. C. Bergey E. J. Maitra A.. (2009). Imaging pancreatic cancer using bioconjugated InP quantum dots. ACS Nano 3, 502–510. 10.1021/nn8008933 19243145 PMC2762404

[B130] Zhang G. Yang Z. Lu W. Zhang R. Huang Q. Tian M.. (2009). Influence of anchoring ligands and particle size on the colloidal stability and *in vivo* biodistribution of polyethylene glycol-coated gold nanoparticles in tumor-xenografted mice. Biomaterials 30, 1928–1936. 10.1016/j.biomaterials.2008.12.038 19131103 PMC2745599

[B131] Zhang H. Yee D. Wang C. (2008). Quantum dots for cancer diagnosis and therapy: biological and clinical perspectives. Nanomedicine 3, 83–91. 10.2217/17435889.3.1.83 18393668

[B132] Zhou Z. Lu Z.-R. (2013). Gadolinium-based contrast agents for magnetic resonance cancer imaging. Wiley Interdiscip. Rev. Nanomed. Nanobiotechnol. 5, 1–18. 10.1002/wnan.1198 23047730 PMC3552562

